# Functional and clinical roles of stromal PDGF receptors in tumor biology

**DOI:** 10.1007/s10555-024-10194-7

**Published:** 2024-07-09

**Authors:** Carina Strell, Elisabet Rodríguez-Tomàs, Arne Östman

**Affiliations:** 1 Department of Immunology, Genetics and Pathology, Uppsala University, Uppsala, Sweden; 2 Centre for Cancer Biomarkers CCBIO, Department of Clinical Medicine, Bergen University, Bergen, Norway; 3 Department of Oncology-Pathology, Karolinska Institutet, Stockholm, Sweden

**Keywords:** PDGF, PDGF receptors, Cancer, Prognosis, Tumor microenvironment, Biomarker, Fibroblasts, Pericytes, Tumor stroma, Response-prediction

## Abstract

PDGF receptors play pivotal roles in both developmental and physiological processes through the regulation of mesenchymal cells involved in paracrine instructive interactions with epithelial or endothelial cells. Tumor biology studies, alongside analyses of patient tissue samples, provide strong indications that the PDGF signaling pathways are also critical in various types of human cancer. This review summarizes experimental findings and correlative studies, which have explored the biological mechanisms and clinical relevance of PDGFRs in mesenchymal cells of the tumor microenvironment. Collectively, these studies support the overall concept that the PDGF system is a critical regulator of tumor growth, metastasis, and drug efficacy, suggesting yet unexploited targeting opportunities. The inter-patient variability in stromal PDGFR expression, as being linked to prognosis and treatment responses, not only indicates the need for stratified approaches in upcoming therapeutic investigations but also implies the potential for the development of PDGFRs as biomarkers of clinical utility, interestingly also in settings outside PDGFR-directed treatments.

## Introduction

Platelet-derived growth factor (PDGF) is a growth factor family exerting important regulatory functions on glial cells and mesenchymal cells such as fibroblasts, vascular smooth muscle cells, and pericytes. Earlier cancer-related investigations into PDGF family members primarily concentrated on the role of oncogenic autocrine PDGF receptor (PDGFR) signaling in, e.g., glioma and sarcoma. However, recent studies on PDGF signaling in cancer have underscored its significant involvement in the reciprocal communication between tumor and stroma cells, influencing cancer progression and, consequently, clinical outcomes.

This review starts with a summary of the molecular biology of the PDGF system, highlighting its developmental and physiological roles. Following this, we discuss experimental tumor biology studies on common solid cancers including breast, lung, prostate, and colon cancer. We also explore analyses of clinical samples to assess the biomarker potential of PDGFRs in solid tumors. Subsequently, therapeutic opportunities are introduced, before concluding with future perspectives outlining key areas for upcoming studies.

## Molecular cell biology of the PDGF system

### PDGF ligands and their receptors

PDGF ligands (PDGF-AA, PDGF-AB, PDGF-BB, PDGF-CC, and PDGF-DD) are disulfide-linked dimers composed of PDGF-A, B, C, and D chains encoded by four genes [1–4]. Two C-terminal distinct splice variants are described for the PDGF-A chain [5]. The ligand dimerization occurs in an anti-parallel manner with receptor-binding parts at each end of the dimer [1, 6, 7]. The long-splice PDGF-A chains, as well as PDGF-B chains, hold a C-terminal cell retention motif restricting ligand exposure to neighboring cells [8, 9]. In contrast to other family members, PDGF-CC and PDGF-DD are secreted as latent dimers to be activated through the removal of autoinhibitory N-terminal domains.

PDGF ligands exert their biological effects through structurally related receptor tyrosine kinases, the PDGF receptor alpha (PDGFRα) and receptor beta (PDGFRβ) [3, 10]. Both receptors are composed of an extracellular region with five Ig-like domains, a single transmembrane helix region, and an intracellular region with a split tyrosine-kinase domain [10]. PDGF ligands bind to the second and third extracellular Ig-like PDGFR domains causing receptor dimerization [11, 12].

**Fig. 1:**
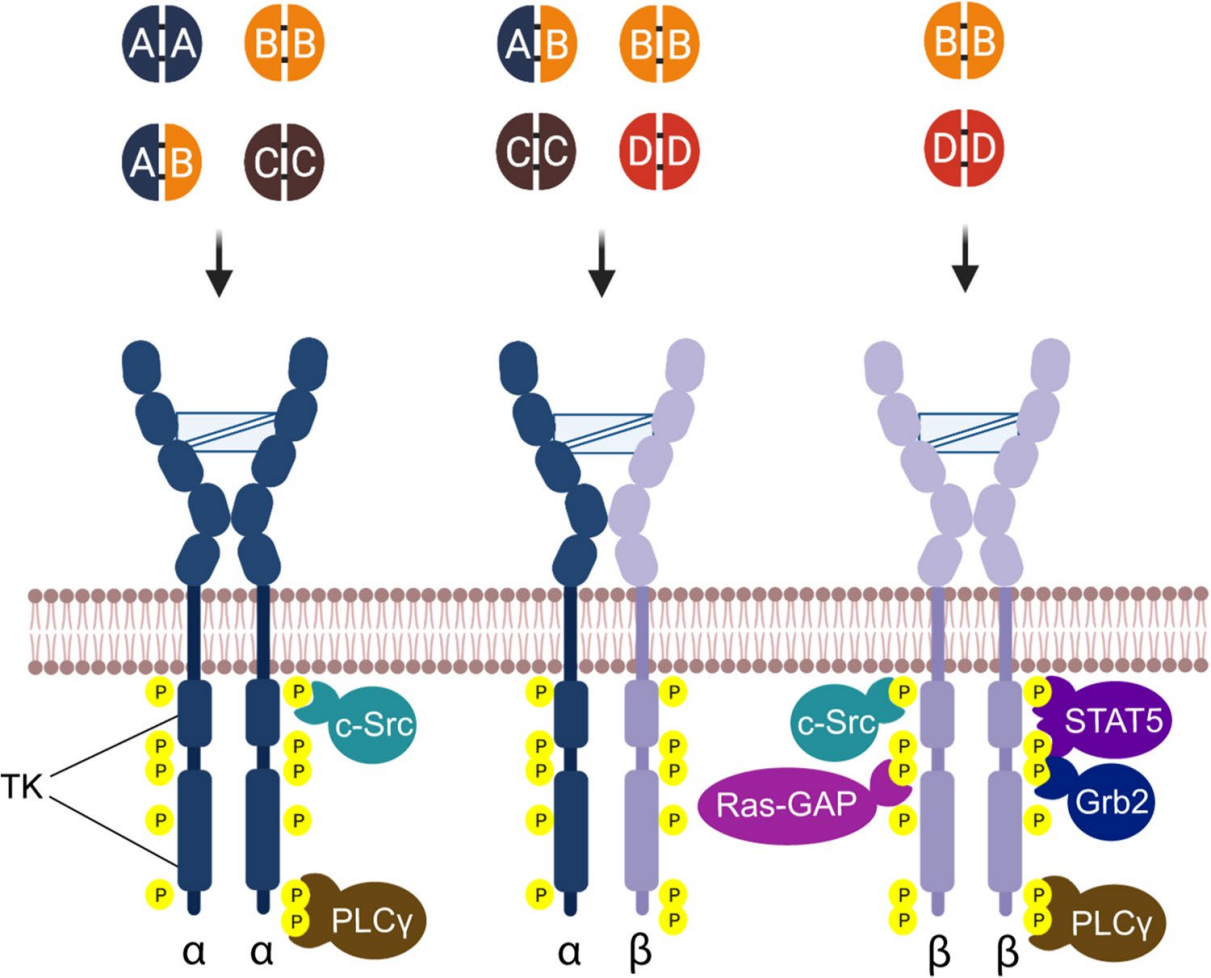
The structural features of the PDGF receptor and ligand interactions. The PDGF receptor is composed of five Ig-extracellular domains, a transmembrane domain, and an intracellular domain. Receptor dimerization occurs through the binding of dimerized ligands (light blue). The PDGF ligand dimers (AA, AB, BB, CC, DD) vary in their receptor specificity (α and β) as shown. Receptor dimerization results in the activation of the kinase domains and phosphorylation of intracellular tyrosine residues, acting as phosphorylation site-specific docking sites for SH2 domain-containing proteins (signaling enzymes (PLC-γ, Src), adaptor proteins (Grb2), and negative regulators (Ras-GAP). The figure schematically highlights signaling proteins common to both receptor homodimers (e.g., Src and PLC-γ) and signaling molecules predominantly linked to one of the homodimers (e.g., STAT5 binding to PDGFRβ homodimers). For additional details, see Heldin review (2013 [11]) and Heldin review (2018 [4]).

The PDGF dimers vary in their receptor specificity (Fig. 1). PDGF-AA, PDGF-AB, PDGF-BB, and PDGF-CC induce PDGFRα homodimers, and PDGF-BB and PDGF-DD are high-affinity ligands inducing PDGFRβ homodimers, while PDGF-AB, PDGF-BB, PDGF-CC, and PDGF-DD are described to induce PDGFRα and β heterodimers [6, 13, 14].

Ligand-induced receptor activation includes receptor dimerization mediated by binding of ligand dimers, which is then further stabilized through receptor-receptor interactions (Fig. 1). This dimerization results in the activation of the kinase domains and phosphorylation of intracellular tyrosine residues acting as docking sites for SH2 domain-containing proteins such as signaling enzymes (PLC-gamma, c-Src), adaptor proteins (Grb2), negative regulators (Ras-GAP), and STAT family members [11, 15]. *In vitro* analyses suggest that PDGFR heterodimers exhibit a considerably reduced autophosphorylation activity compared to homodimers [16, 17].

Mechanisms for negative regulation or termination of PDGFR signaling include ligand/receptor internalization, ubiquitin-dependent degradation, and de-phosphorylation by tyrosine phosphatases including PTP1B, PTP-2C, or SHP2 [4, 18–22].

PDGF signaling exerts context-dependent signaling interactions. Well-documented cellular responses to PDGF stimulation include cell proliferation, chemotaxis, and contraction of collagen matrixes [18].

## Developmental, physiological, and pathophysiological roles of PDGF

Analyses of global or cell-type-specific PDGF ligand or receptor knockout mice have identified developmental processes controlled by PDGFs/PDGFRs in an isoform-specific manner. A common theme emerging from these studies is niche-specific paracrine signaling with PDGF ligands produced by the epithelial/endothelial cells that stimulate the recruitment and proliferation of juxtaposed PDGFR-expressing mesenchymal cells.

Of note, there is only limited published evidence regarding the *in vivo* presence and function of PDGFRα and β heterodimers, with one study having suggested a potential role of heterodimers in craniofacial development [23].

### Epithelial-mesenchymal interactions involving PDGF-AA, PDGF-CC, and PDGFRα

Mesenchymal PDGFRα signaling has been shown to be of importance for proper development in a series of organs, including the lung, the GI tract, the skin, the testis, and the CNS. Regarding the main PDGFRα ligands, the different phenotypes following the knockout of *Pdgfc* and *Pdgfa* suggest tissue-specific effects of PDGF-AA and PDGF-CC [24, 25].

Lungs of PDGF-A knockout mice show morphologically abnormal, enlarged alveoli with an emphysema-like phenotype. This phenotype includes the absence of PDGFRα-positive mesenchymal cells in the walls of alveolar saccules [26, 27]. PDGFRα-positive fibroblasts include airway smooth muscle cells and interstitial myo- as well as matrix-fibroblasts. This lung phenotype suggests PDGFRα-dependent instructive functions of the mesenchymal cells required for proper epithelial and tissue development. *Pdgfa* knockout mice also show reduced formation of intestinal villi, associated with loss of pericryptal mesenchyme [28]. Candidate PDGFRα-dependent mesenchymal regulators of epithelial phenotypes include Wnt ligands and R-spondins [29, 30].

Additional tissues where PDGFRα signaling contributes to proper development and regeneration include the skin [31], postnatal white adipose tissue [32], and skeletal muscle [33]. PDGFRα signaling is also important in CNS, where it controls the migration of oligodendrocyte progenitors [34, 35].

Concerning pathological processes, fibrotic processes such as pulmonary or dermal fibrosis [36–39] as well as obesity-induced white adipose tissue fibrosis [40] were found to be associated with deregulation of the PDGFRα axis. Additional pathologies where PDGFRα has been implied include inflammatory bowel disease, where PDGFRα-positive mesenchymal niche cells perturb epithelial proliferation and maturation [30].

### Blood vessel–mural cell interactions involving PDGF-BB, PDGF-DD, and PDGFRβ

PDGF-B and PDGFRβ are expressed by endothelial cells and adjacent mural cells, respectively, and the phenotypes of *Pdgfrb* and *Pdgfb* knockout mice strongly support critical roles of a PDGF-BB/PDGFRβ endothelial/mural cell axis for the recruitment of vascular mural cells and associated vessel function (reviewed in [6, 41, 42]).

Both knockout models are lethal and display microvascular bleedings associated with reduced pericyte coverage, endothelial hyperplasia, and abnormal capillary morphogenesis [43–47]. Short-range interactions are suggested by findings that deletion of the C-terminal retention motif is sufficient to cause pericyte detachment from microvessels [48]. Subsequent studies have demonstrated variations between tissues regarding the strength of the phenotype indicating alternative mechanisms for proper endothelial/mural cell interactions.

The relevance of the PDGF-BB/PDGFRβ axis for vascular function has also been supported by human genetics, through the identification of vascular defects associated with loss-of-function mutations of PDGF-B and PDGFRβ in familial idiopathic ganglia calcification (reviewed in [49]).

In agreement with important roles in vascular biology, PDGFRβ signaling has been proposed to contribute to various cardiovascular diseases including atherosclerosis [50, 51], restenosis [52], stroke [53], aortic aneurysm [54, 55], and pulmonary hypertension [56, 57].

Collectively, these studies underscore the coordinated recruitment of PDGFR-expressing mesenchymal cells to distinct tissue niches, facilitated by specific PDGF ligands produced by epithelial or endothelial cells. Disruptions in these niches are linked to pathological states, suggesting that perturbed PDGF signaling plays also a role in tumor biology.

## Tumor phenotypes and PDGFR signaling

As detailed below, a series of different effects of PDGFRs on fibroblasts and pericytes has been proposed to positively or negatively affect tumor growth.

### Effects of PDGFRs on tumor initiation and growth

**Fig. 2:**
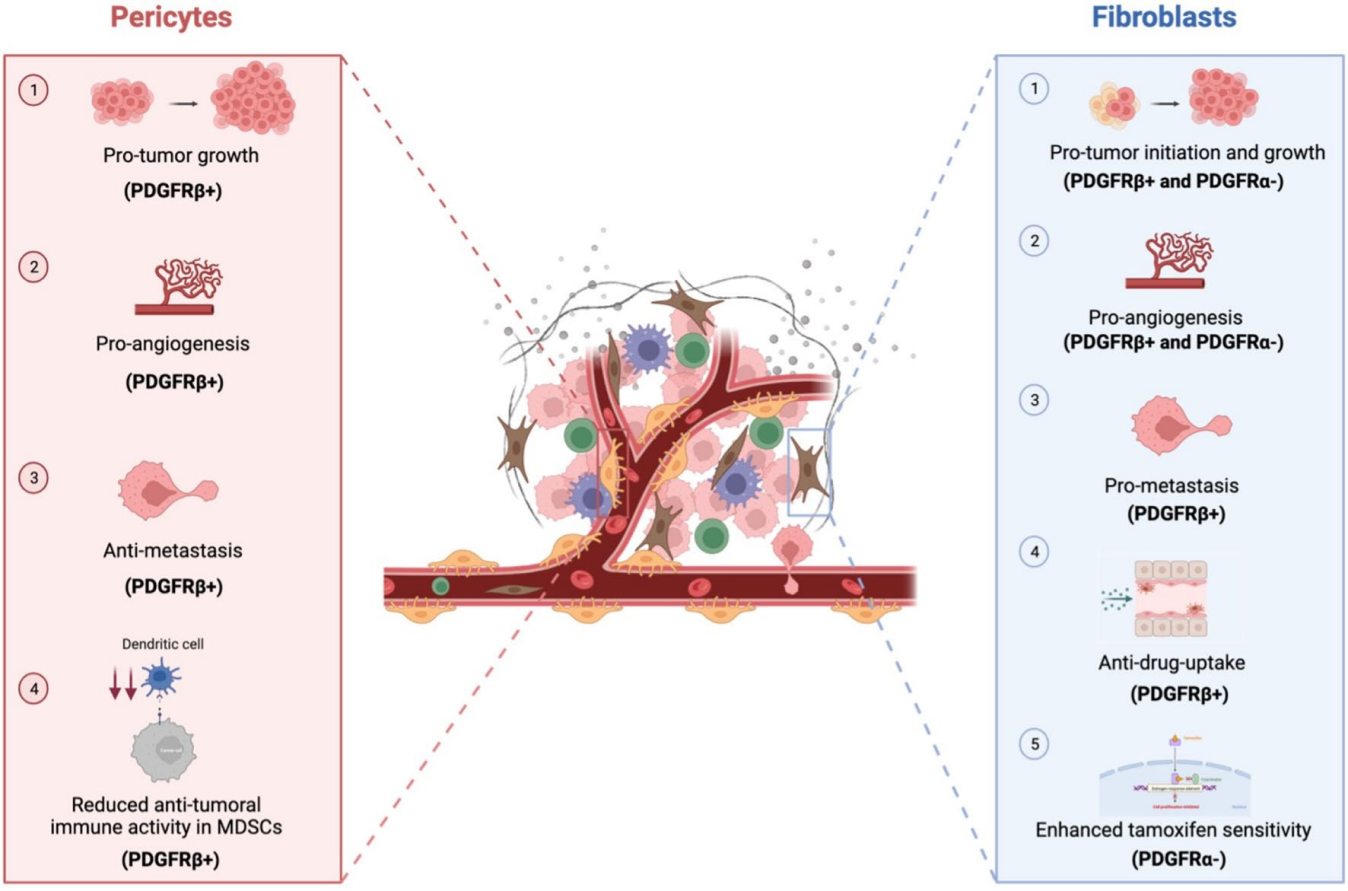
The impact of PDGFRs on pericytes and fibroblasts. The main effects of PDGFRs (α and β) on pericytes (left) and on fibroblast (right), supporting or inhibiting tumor growth and drug resistance are shown

Tumor-stimulatory effects of paracrine PDGF signaling were first suggested in mouse studies with PDGF-BB over-expressing melanoma cells, negative for PDGFRs [58]. Corresponding histologic analyses proposed that the growth advantage was related to increased angiogenesis and recruitment of tumor-supportive fibroblasts (Fig. 2 upper right (1 and 2)). Follow-up studies in other tumor models of, e.g., skin, breast, and lung cancer with PDGFR-negative malignant cells also demonstrated pro-tumoral effects mediated by different PDGF ligands that were associated with increased fibroblast recruitment [59–63]. Although not conclusive regarding underlying mechanisms, these studies preliminary identified PDGFR-dependent fibroblast-derived secreted factors including FGF2 and 7 [64], CXCL12 [65], or HGF [66]. In line with those findings, pharmacological inhibitors of PDGFR signaling showed therapeutic effects in a genetic mouse model of cervical cancer [64].

In addition to these studies above, mostly implying PDGFRβ as the key receptor, other studies have linked PDGFRα to tumor-restraining effects. Analyses of clinical samples and model systems of breast DCIS identified an epithelial cell-induced re-programming of mesenchymal cells leading to downregulation of PDGFRα. This occurred via Notch signaling, particularly at sites of basement membrane disruptions [67]. Another study, also linking loss of PDGFRα-positive cells to tumor progression, reported the accumulation of PDGFRα-negative bone marrow–derived fibroblasts at the primary tumor as well as the metastatic sites of the MMTV-PyMT transgenic mouse breast cancer model [68]. The PDGFRα-negative fibroblasts enhanced tumor growth and angiogenesis (Fig. 2 upper right (1 and 2)). Single-cell sequencing of fibroblasts from this model confirmed the presence of PDGFRα-negative and PDGFRα-positive fibroblast populations with gene expression signatures associated with angiogenesis and matrix remodeling, respectively [69].

The importance of PDGFRβ on pericytes for tumor growth in dependence on angiogenesis has also been analyzed in multiple mouse models (Fig. 2 upper left (1 and 2)). Experiments using the hypomorphic PDGFB-ret/ret mouse model, which expresses a truncated form of PDGF-B, lacking the C-terminal retention motif, resulted in impaired tumor angiogenesis and reduced pericyte coverage implying PDGF-BB/PDGFRβ as a promising, potential therapeutical target [70]. Accordingly, overexpression of PDGFRβ ligands in mouse melanoma cells induced a pro-tumoral pericyte-rich vascular phenotype [71]. Furthermore, mouse model studies combining VEGFR- and PDGFRβ-inhibition support continued studies on the targeting of PDGFRβ-positive perivascular cells [72–74]. PDGFR inhibitors have also been used in mouse models to demonstrate roles for PDGFR signaling in pro-tumoral vascular mimicry mediated in part by the recruitmet of pericytes [75].

### Effects of PDGFR signaling on metastasis

Pro-metastatic effects of PDGF-activated fibroblasts have been observed in different mouse models (Fig. 2 right half (3)). A significant reduction of metastasis, without major effects on primary tumor growth, was observed in colon cancer models using the receptor tyrosine inhibitor imatinib mesylate, which blocks both PDGFRs among other tyrosine kinases [76]. Support for this concept, and some mechanistic details, was added in a study showing that PDGF-BB-stimulated fibroblasts enhance the migratory and invasive capacities of colorectal cancer cells in a manner involving secretion of PDGF-induced Stanniocalcin-1 (STC1) [77]. Furthermore, PDGF-BB-stimulated fibroblasts exhibited pro-metastatic capacities already at the early stages of different tumor types using a co-implantation zebrafish model for cancer metastasis [78].

The approach of suicide gene–mediated depletion of PDGFRβ-positive pericytes has been used to analyze the impact of PDGFRβ-positive pericytes on tumor metastasis (Fig. 2 bottom left (3)) [79, 80]. In both studies, loss of PDGFRβ-positive perivascular cells induced a pro-metastatic vascular phenotype, including increased hypoxia, c-Met-dependent tumor cell stimulation, and increased angiopoietin-2-dependent angiogenesis. However, as PDGFRβ is expressed in other mesenchymal cells, a potential contribution of non-pericyte cells to the observed phenotype cannot be entirely excluded. In line with those studies, other work indicated that highly aggressive tumor cells often express elevated levels of PDGF-BB, promoting pericyte proliferation within the tumor microenvironment and facilitating vascular remodeling and ultimately tumor growth. Interestingly, corresponding mice models further proposed that an inhibition of PDGFRβ signaling with blocking antibodies was only beneficial in specific tumors with high PDGF-BB levels. In contrast, in tumors with low PDGF-BB levels, this inhibition led to pericyte loss from vessels, resulting in increased tumor dissemination and metastasis rather than suppression [75, 81].

Two recent studies have also suggested the particular importance of stromal PDGFRβ signaling for brain metastasis using both genetic mouse models and pharmacological approaches [82, 83]. Notably, these effects possibly include stromal PDGFRβ in the primary tumors, as well as in the astrocytes of the metastatic tumor microenvironment [82]. Regarding regulatory mechanisms, one of these studies implied tumor epithelial-derived HIF1 activation as a driver of the production of pro-metastatic PDGFRβ ligands [83].

Regarding key signaling components for stromal PDGFRβ-induced metastasis details remain, in general, to be defined. However, based on combined analyses of cell models, mouse studies, and analyses of clinical samples, integrin α11 was identified as a critical partner for PDGFRβ-induced metastasis [84].

Similarly, cell types most important for stromal PDGFRβ-induced metastasis are not yet fully defined. Interestingly, platelet-specific deletion of PDGF-B reduced experimental metastasis in multiple models, indicating this cell type is an important provider of ligands [85]. Other implied cell types include endothelial cells and macrophages [86, 87]. Additionally, MDSCs in the metastatic niche have been suggested as an important producer of pro-angiogenic PDGF-BB in a CXCL17-dependent manner (Fig. 2 bottom left (4)) [88].

### Effects of PDGFR signaling on tumor interstitial fluid pressure and drug uptake

Pivotal observations in edema models suggested important effects of PDGFRβ signaling in fibroblasts on interstitial fluid pressure (IFP) (reviewed in [89]).

These findings prompted tumor model studies exploring the concept that PDGFRβ targeting could be an approach to reduce the typically high IFP in tumors, allowing for increased uptake and efficacy of systemically delivered drugs. In studies with different tumor models, PDGFR inhibition was indeed found to reduce tumoral IFP, thereby improving drug uptake and efficacy (Fig. 2 bottom right (4)) [90–93]. Notably, PDGF inhibitors increased the efficacy of various types of drugs including standard chemotherapy agents such as 5-FU and Taxol, as well as radiolabeled tumor-targeted antibodies.

### PDGFRα-mediated regulation of ER status and tamoxifen sensitivity

An innovative and original study analyzing breast cancer growth in PDGF-CC knockout mice unraveled unexpected PDGFRα-dependent control of ER expression and tamoxifen sensitivity in breast cancer (Fig. 2 bottom right (5)) [94]. This study identified a pathway where fibroblast-derived PDGF-CC induced a basal-like epithelial phenotype, which could be blocked by PDGF-CC antibodies. Notably, this converted the epithelial cells to an ER + phenotype and conferred sensitivity to tamoxifen. HGF, previously associated with the induction of a basal-like epithelial/tumor cell fate, was suggested as a key mediator of the adverse effects of PDGF-CC-activated fibroblasts.

Taken together, despite extensive research on PDGF signaling in tumors, our understanding of its precise roles in tumor biology remains incomplete, and the molecular downstream mediators described have not yet advanced as therapeutic targets. This is despite the well-established role of PDGF signaling in development and can be attributed in part to the complexity of tumor heterogeneity, encompassing both genetics and cellular composition. While *in vitro* tissue cultures and mouse tumor models have been valuable, they have limitations in capturing this heterogeneity and may thus not fully represent the diverse aspects of PDGF signaling. Future studies should aim to integrate tissue-based analyses of large, well-defined patient cohorts with supplementary functional studies. Such an approach could refine the discovery of clinically relevant and targetable aspects of PDGF signaling in tumor biology.

## PDGFRs as prognostic and predictive biomarkers

Given the strong preclinical evidence for PDGF signaling as an important regulator of fibroblasts and perivascular cells in the tumor microenvironment during tumor progression, multiple studies investigating the potential of PDGFRs as prognostic as well as predictive biomarkers in different tumor types have been conducted over the past decade (an overview is presented in Table 1).

**Table 1:** Selected studies linking stromal PDGFRs to prognosis and response to treatment

**Studies linking stromal PDGFRs to poor prognosis**						
*Study (year)*	*Tumor type*	*Samples* (*n*)*^1^	*Technique*	*Localization*	*Markers*	*Ref*
Paulsson et al. (2009)	Breast	507	IHC	Stromal overall	High PDGFRβ expression, multivariable: *p* = 0.02^§^	[95]
Bartoschek et al. (2018)	Breast	768	Gene signature	-	Vascular CAFs (vCAFs) gene signature, multivariable: *p* = 0.001^Ϯ^	[69]
Frings et al. (2013)	Breast	*Cohort 1:* 159 *Cohort 2:* 295 *Cohort 3:* 286	PDGF gene signature	-	High PDGF signature score, multivariable: *Cohort 1*: *p* = 0.036^§^; *Cohort 2*: *p* = 0.031^§^; *Cohort 3*: *p* = 9.51 × 10^−4§^	[96]
Strell et al. (2021)	Breast	989	IHC	Stromal overall	Medium PDGFRβ expression, multivariable: *p* = 0.042^§^	[97]
Corvigno et al. (2016)	Ovarian	186	IHC	Tumor stroma and perivascular area	High PDGFRβ-positive tumor stroma fraction, multivariate: *p* = 0.01^¥^, and high PDGFRβ-positive perivascular intensity, multivariable: *p* = 0.03^¥^	[98]
Sun et al. (2016)	Thyroid papillary carcinoma	339	IHC	Stromal overall	High PDGFRβ expression, univariable: *p* = 0.035^¥^	[99]
Hägglöf et al. (2010)	Prostate	377	IHC and IF	Stromal overall	High PDGFRβ expression, univariable: *p* < 0.001^ł^	[100]
Nordby et al. (2017)	Prostate	535	IHC	Stromal overall	High PDGFRβ expression, multivariable: *p* = 0.010^§Ϯ^	[101]
Frödin et al. (2017)	Renal cell carcinoma	287	IHC	Perivascular area	High PDGFRβ expression, multivariable: *p* = 0.009^¥^	[102]
Ehnman et al. (2013)	Rhabdomyosarcoma	177	IHC	Tumor stroma	High PDGFRβ expression, univariable: *p* = 0.0032^Ϯ^	[103]
Kilvaer et al. (2019)	Non-small cell lung cancer	*Cohort 1:* 553 *Cohort 2:* 367	IHC	Stromal overall	High PDGFRβ expression, multivariable: *p* = 0.020 (*Cohort 1*^ł^* and 2*^¥^) High PDGFRα expression, multivariate: *p* = 0.016^ł^ (*Cohort 1)* and *p* = 0.010^ł^ (*Cohort 2*)	[104]
Pellinen et al. (2022)	Non-small cell lung cancer	*Cohort 1:* 318 *Cohort 2* 318	Multiplex IF and mRNA expression	Stromal overall	High PDGFRA-/PDGFRB + /FAP + /αSMA + expression, univariable, *p* = 0.0092^¥^ (*Cohort 2*), *p* = 0.02^¥^. Low PDGFRA + /PDGFRB + /FAP-/αSMA + expression, (*cohort 1*), *p* = 0.018^¥^ *(Cohort 2*), *p* = 0.016^¥^ (C*ohort 1*) High FAP to PDGFRA gene expression ratio, multivariable: *p* < 0.01^¥^*^2^	[105]
Lin et al. (2020)	Oral squamous cell carcinoma	149	PDGF and PDGFR mRNA expression levels	-	High PDGFRA, PDGFB, and PDGFRB, multivariable: *p* < 0.001^¥^	[106]
Han et al. (2021)	Esophageal squamous cell carcinoma	179	PDGFA mRNA expression levels	-	High PDGFA mRNA expression, multivariable: *p* = 0.034^¥^	[107]
**Studies linking stromal PDGFRs to poor response to treatment**						
*Study (year)*	*Tumor type*	*Samples* (*n*)*^1^	*Technique*	*Localization*	*Markers*	*Ref*
Strell et al. (2021)	Breast	989	IHC	Stromal overall	No significant interaction between RT and PDGFRβ score*^3^	[97]
Paulsson et al. (2016)	Breast	*Cohort 1:* 500 *Cohort 2:* 912	IHC	Stromal overall	High stromal PDGFRβ expression*^4^	[108]
Strell et al. (2021)	Breast (DCIS)	540	IHC	Stromal overall	High PDGFRβ expression, multivariable formal interaction test between PDGFRβ and RT: *p* = 0.040*	[109]

Larger sample size (more than 50), human studies, and preference for multivariable rather than univariable analyses were the inclusion criteria used to include studies in the table

^1^Number of samples associated with the follow-up data (patient’s prognosis and response to treatment) or clinical data

^2^Bulk mRNA expression data from an external lung cancer dataset (*n* = 1925) was used for survival analysis

^3^The benefit of RT regarding the risk of ipsilateral breast tumor recurrence (IBTR) was significant in low and medium PDGFRβ expression groups, multivariable *p* = 0.004 and *p* < 0.001, respectively, but not in high PDGFRβ expression group, multivariable, *p* = 0.110

^4^A significant benefit of tamoxifen treatment was detected in the low/moderate PDGFRβ expression pre-menopausal group (*Cohort 1*: *p* = 0.026), but it was not seen in the high PDGFRβ expression group (*p* = 0.510)

^§^Recurrence-free survival

^Ϯ^Development of metastatic disease

^ł^Disease-specific survival

^¥^Overall survival

^*^Ipsilateral breast events

A first major description of the prognostic relevance of stromal PDGFRβ expression was published on invasive breast cancer through conventional IHC analysis of a population-based cohort of more than 200 cases and uncovered significant associations between high stromal expression and shorter recurrence-free and breast cancer–specific survival based on univariate analyses [95]. This occurred together with strong positive correlations between stromal PDGFRβ and poor prognosis markers such as high grade, high proliferation, and HER2 amplification. Subsequent studies of additional breast cancer cohorts including samples derived from randomized clinical trials have provided overall support for these findings [97, 108, 110].

Similar associations between high stromal PDGFRβ expression and poor prognosis have also been demonstrated, again by conventional IHC analyses, for other solid cancer types including prostate, gastric, colorectal, pancreatic, oral squamous cell, and thyroid papillary cancers [99–101, 106, 111–114] as well as rhabdomyosarcoma, with the latter indicating that stromal PDGFRβ is also relevant for prognosis in soft tissue sarcomas [103].

Given the role of PDGFRβ as a key regulator of pericytes, dual IHC stainings with automated quantitative scoring approaches have been established, which enable an in-depth analysis of perivascular PDGFRβ status and its associations with vascular features as well as clinical patient characteristics. Significant associations between poor prognosis and specifically high perivascular PDGFRβ expression have been noted in high-grade serous ovarian cancer and kidney cancer, which were maintained in multivariate analyses [98, 102]. Additionally, a study on a moderately sized (*n* = 36) cohort of oral squamous cell carcinoma, which investigated the clinical relevance of pericyte-related genes, indicated a good prognosis association of stromal PDGFRβ based on gene expression analyses [115].

The potential of PDGFRβ as a predictive biomarker for treatment benefit has first been demonstrated in an IHC-based *post hoc* analysis of two randomized trials for adjuvant tamoxifen treatment of early breast cancer including either pre- or post-menopausal women [108]. In the pre-menopausal cohort, patients with low stromal PDGFRβ expression displayed a significant benefit of tamoxifen regarding recurrence-free survival, while no significant effects were noted for patients with high PDGFRβ expression. Interestingly, in the post-menopausal cohort, significant results in the same directions were only obtained when analyses were restricted to cases with > 75% ER positivity.

Further evidence for PDGFRβ as a clinically relevant predictive biomarker has been obtained from *post hoc* analyses of tissue collections from two randomized adjuvant radiotherapy trials in DCIS and stage I/IIA breast cancer [97, 109]. In both studies, the benefit from radiotherapy with regard to disease recurrence was restricted to women with low PDGFRβ expression in the stroma of the primary tumor. A strong significant interaction between stromal PDGFRβ and radiotherapy was however only confirmed in adjusted formal interaction tests within the DCIS cohort. Encouraged by these studies, continued efforts are expected in the upcoming years, addressing the role of PDGFRβ as a biomarker in tumor collections derived from radioimmunotherapy trials.

Compared to PDGFRβ, less data is available regarding the role of PDGFRα as a potential prognostic or predictive biomarker. Single-cell sequencing of cancer-associated fibroblasts from a genetic murine breast cancer model suggested that high PDGFRα expression marks a resting, local fibroblast population [69]. IHC analysis of two DCIS cohorts demonstrated indeed good prognosis trends for stromal PDGFRα and could identify a significant poor prognosis association, particularly for cases with high PDGFRβ expression combined with low PDGFRα level [67]. In line with those findings, good prognosis associations for stromal PDGFRα have further been independently described in invasive breast cancer [68]. Only very few studies have addressed the role of stromal PDGFRα in other solid tumors; analyses of two population-based non-small lung cancer cohorts have also identified associations between high PDGFRα and good prognosis using conventional IHC; however, this finding was restricted to cases with squamous cell carcinoma histology [104]. Independent support for this observation was given in a recent study using multiplex fluorescent immunohistochemistry to analyze four different fibroblast markers simultaneously also within two TMA collections of well-annotated population-based non-small lung cancer cohorts [105]. Among the 15 different marker-defined fibroblast subsets investigated in this study, it was noted that specifically PDGFRα^+^/PDGFRβ^+^/ASMA^+^/FAP^−^ fibroblasts and PDGFRα^−^/PDGFRβ^+^/ASMA^+^/FAP^+^ fibroblasts showed opposite associations with clinicopathological characteristics, with PDGFRα^+^/PDGFRβ^+^/ASMA^+^/FAP^−^ fibroblasts exhibited negative associations with squamous histology and immune suppressive features but positive associations with EGFR mutations and good prognosis. Importantly, the study highlighted multiplexed marker profiling as a powerful tool to identify clinically relevant cellular subsets [105].

In the context of the described good prognosis associations of PDGFRα in solid tumors, it is surprising to note that analyses of the receptor’s ligand PDGF-AA indicate poor prognosis associations for oral squamous cell carcinoma, ovarian cancer, sarcoma, and esophageal squamous cell carcinoma [106, 107, 116, 117]. Those analyses were based on crude mRNA expression levels and did not yet discriminate between the described alternative splice variants of PDGF-A, leading to either soluble or matrix-retained PDGF-AA protein.

In summary, a high stromal PDGFRβ expression appears to be consistently associated with poor prognosis and reduced treatment benefit, while PDGFRα in contrast is rather associated with good prognosis.

Future studies investigating the potential of PDGFRs as clinical biomarkers are likely to benefit from recent advances in highly multiplexed spatial tissue profiling techniques. Those novel tools represent valuable resources in biomarker research, as they allow to quantitate subsets of PDGFRα- and/or PDGFRβ-positive cells specifically as well as in combination with other fibroblast markers as suggested by emerging single-cell datasets. Signals might further be enhanced through the integration of spatial information in scoring algorithms such as the relative position of PDGFR-positive cells to, e.g., epithelial or immune cells.

Nevertheless, it should be noted that IHC approaches monitoring PDGFR protein in general do not allow conclusions regarding receptor activation status and thus active PDGF signaling. While phospho-PDGFR antibodies exist and have been successfully applied in cell culture-based studies [118], the specificity of these antibodies in diagnostic FFPE samples has raised concern. Proximity ligation assays for the detection of phosphorylated PDGFRs have also been described [119–121], but have not yet been used to report on stromal PDGFR status in clinical samples. A common concern with methods detecting phosphorylated, and thus active, PDGFRs in tissue sections is their susceptibility to artifacts caused by tissue handling and fixation techniques.

An alternative, bioinformatical approach to investigate PDGF signaling activity in clinical tissue samples was developed in the form of a “PDGFRβ-signature,” derived from PDGF-BB-activated, cultivated fibroblasts [96]. Analysis of gene expression datasets of four different breast cancer cohorts showed consistent associations between high signature scores and poor prognosis in multivariable analyses with recurrence-free survival or disease-specific survival/overall survival as endpoints. Importantly, significant prognostic associations remained in comparison to other stroma-related signatures [96].

In this context, it should be highlighted that improved approaches to analyze PDGFR activation status in diagnostic tissue samples are of the highest interest and clinical relevance, given the emerging recognition and development of therapeutical strategies targeting active PDGFRs to overcome cancer progression and therapy resistance as discussed in the next chapter. Such technical advances can possibly be achieved by assays specifically detecting receptor-adaptor protein complexes such as PDGFRβ-Grb2 or PDGFRb-PI3K through proximity ligation assays [122] or, alternatively, by multi-probe *in situ* RNA profiling for PDGFR activation-associated target genes.

## Therapeutic opportunities

The therapeutic targeting of stromal PDGFR signaling in selected patient populations is encouraged by findings from the experimental and correlative studies as outlined above.

Indeed, different strategies have been considered for blocking PDGF signaling in the therapeutical, clinical setting. Small molecule inhibitors are available (e.g., imatinib); however, their specificity remains a common problem, given the high similarity in the catalytic domains of receptor tyrosine kinases such as PDGFRs, c-Kit, Abl or Flt3; consequently, this represents a major challenge when interpreting trial results from studies using small molecule inhibitors to block receptor tyrosine kinases.

Although motivated by a series of preclinical studies that showed tumor growth and progression restraining effects of imatinib in different breast cancer models [123–127], clinical trials could not demonstrate consistent and strong clinical efficacy of imatinib or other non-selective PDGFRβ inhibitors in breast cancer, as well as several other solid cancer types (reviewed in detail in [128–130]). Importantly, the clinical efficacy of imatinib could not be refined by selection of a patient subset with high stromal PDGFRβ expression, suggesting a need for better patient stratification based on actual active tumoral PDGF signaling [131]. This need was further underlined in an elegant preclinical study using a transgenic knock-in mouse strain carrying a silent mutation allowing a specific inhibition of PDGFRβ signaling through the compound 1-NaPP1 [132]. The tumor-inhibiting efficacy of the compound was indeed restricted to models expressing both high PDGFB as well as high stromal PDGFRβ levels, highlighting the importance of improved knowledge on active tumoral PDGF signaling for future clinical applications.

As discussed previously, PDGFRβ signaling in pericytes has been implicated in both tumor growth and metastasis [71, 73, 75, 79–81, 133], suggesting potential therapeutic opportunities to be further explored. One important consideration would therefore be to compare and contrast combined PDGFRβ-VEGFR targeting versus mono-treatment. Based on the present data situation, it can be envisioned that the different approaches will show different efficacy depending on the context including even combined use with chemotherapy or immune-stimulatory drugs.

Preclinical *in vitro* and *in vivo* data on the immunomodulatory effects of receptor tyrosine kinases have been conflicting, likely because of the complex and paracrine nature of intratumoral immune regulation. However, promising first results for famitinib (PDGFR/c-kit/VEGFR inhibitor) in the context of immunotherapy were very recently reported from an open-label, single-arm, phase II study including patients with untreated, advanced, immunomodulatory triple-negative breast cancer, who received a novel combination of famitinib, camrelizumab (monoclonal antibody against PD-1) and nab-paclitaxel [134]. The corresponding randomized controlled trial is initiated to follow up on these findings, and it will be interesting to see analyses specific to subsets with high stromal PDGF signaling activity and/or PDGFRβ expression.

Attempts to block signaling via the two PDGFR isoforms more specifically are emerging. For PDGFRα blocking, monoclonal antibodies have been developed [135, 136] and tested in clinical trials of soft tissue sarcomas, showing promising results in phase II studies [137]. Nevertheless, no correlation between general PDGFRα expression and outcome was detected [138]. Furthermore, the promising results from the phase II study were not reproduced in a larger phase III study [139].

Specific PDGFRβ inhibitors tailored for oncology have not yet entered clinical trials. A nuclease-resistant RNA aptamer (Gint4.T) was recently described as a high-affinity inhibitor of PDGFRβ in mouse models of glioblastoma and metastatic triple-negative breast cancer, showing tumor-suppressive effects [140, 141]. Preclinical *in vitro* and *in vivo* models of triple-negative breast cancer provided further promising data regarding reduced tumor growth and metastasis in combinatorial treatment studies with the PDGFRβ-inhibiting aptamer and anti-PD-L1 antibodies [142]. It is noted that both, preclinical studies and early trials, suggest a potential benefit from co-targeting PDGFRβ and immune checkpoints. This approach may hold promise, particularly for triple-negative breast cancers, which frequently show elevated expression levels of both PD-L1 and stromal PDGFRβ [110, 143].

Another example of a patient subset-specific effect of PDGFRβ inhibition was recently provided in a preclinical study using BRCA1-deficient breast cancer models, where targeted deletion of *Pdgfrb* suppressed tumorigenesis [144]. However, validation studies and clinical evidence for this observation are still missing. Nevertheless, in line with previous studies, one can conclude that there are indications that the therapeutical efficacy of PDGF signaling inhibition might further depend also on differences in the underlying fibroblast biology as for example fibroblast subset composition within tumors of different histologic or genetic background. In consequence, experimental models for therapeutical studies need to be carefully chosen, and *post hoc* analyses of clinical trials within specific patient subgroups are highly motivated.

A different, but very promising therapeutic approach, which raised attention during the past decade, uses nanocarriers with a cyclic peptide binding specifically to PDGFRβ (reviewed in [145]) or PDGFRβ affibodies for the specific delivery targeting agents to the tumor site (referred to as “homing”). Preclinical mouse model example studies have demonstrated antitumoral efficacy with this approach by fusing a PDGFRβ affibody with immunomodulatory agents such as TRAIL [146] or immune checkpoint-blocking antibodies [147]. PDGFRβ affibodies combined with radiotracers have also been described to improve PET imaging [148], which raises the interesting future possibility for PDGFR profiling of metastatic lesions using non-invasive modalities.

Finally, in drug repurposing studies, the antidiabetic agent metformin, a member of biguanides, was found to show antitumor proliferative and metastatic activities. While the mechanism of action remains poorly understood, one study using a triple-negative breast cancer mouse model linked metformin to a reduced expression of PDGF-BB in tumor cells and consequently vessel normalization and reduced lung metastasis [149]. Given increasing efforts in drug-screening studies aiming to interrupt the paracrine tumor-stroma crosstalk during cancer progression, potentially further components disrupting or targeting paracrine PDGF signaling in cancer might be uncovered.

## Future perspectives

As presented in previous sections, mechanistic studies strongly suggest that PDGFRs exert important and differential regulatory functions impacting solid tumor behavior. Furthermore, tumor profiling studies are uncovering inter-case variability, where stromal PDGFR expression is associated with prognosis and response to treatment. The following paragraphs suggest a few areas that are believed to be productive for advancing present findings to a stage, where the PDGF system is exploited as biomarkers of clinical utility or drug targets.

Regarding tumor biology, emerging findings suggest differential functional effects of PDGFRα and PDGFRβ homodimers. In this context, the recent advancement of proximity ligation assays for tissue analyses appears as a promising tool to fill our knowledge gap on the prevalence and role of PDGFRα and PDGFRβ heterodimers in the spatial tissue context. The well-documented inter-case variability in PDGFR expression levels prompts continued analyses to identify the key mechanisms for the regulation of stromal PDGFR expression. The findings that fibroblasts exert important immune regulatory roles (reviewed in [150–152]) suggest additional studies analyzing how PDGFR signaling influences the immune-modulatory effects of fibroblasts. Similarly, findings that fibroblast subsets control normal stem cells and cancer stem cells (reviewed in [153, 154]) motivate further specific studies on the involvement of PDGFRs in these processes.

Specific analyses of the roles of stromal PDGFRs in sarcoma remain a promising and possibly fruitful area, with advancing methodologies that enable the distinction between stromal and malignant sarcoma cells. Hopefully, a combination of experimental studies and tissue analyses may further elucidate the functional significance of stromal PDGFRs in sarcoma [103].

While this review focused on PDGFRs in solid cancers, PDGFR signaling plays also a significant role in hematological cancers, such as leukemia and lymphoma, regulating cell growth, survival, and differentiation. Here, the aberrant activation of PDGFR signaling is often due to genetic mutations, particularly fusion events [155]. However, its involvement in the microenvironmental regulation of bone marrow niche-dependent hematological malignancies remains relatively unexplored and warrants further investigation in future studies.

As for continued efforts to develop PDGFR-related biomarkers of clinical utility, it is obvious that existing findings, dominated by conventional single-marker IHC, need to be further validated in additional high-quality cohorts. It is envisioned that continued efforts to score and quantify subsets of PDGFR-positive fibroblasts, as defined by additional markers and/or localization, will improve the strengths of prognostic and response-predictive signals. An improvement in the biomarker field would thereby be achieved through the implementation of methods, as discussed above, allowing determination not only of PDGFR expression but also of PDGFR activation. Finally, it is noted that PDGFR status in metastasis, and the associated biomarker potential, remains largely unexplored, with some emerging studies suggesting clinically and mechanistically relevant between-case variability [156].

Targeting of PDGFRs remains a promising approach. A series of limitations of previous efforts should be considered in upcoming efforts. The indications of differential function and expression of PDGFRα and PDGFRβ strongly suggest the development of isoform-specific agents both for inhibitory targeting and use as payload delivery. Furthermore, the heterogeneity in expression highlights the need for proper patient selection, possibly requiring novel imaging-based tools for monitoring PDGFR status.

Altogether, there are thus reasons to remain optimistic that PDGFR studies performed until now will ultimately appear productive from the perspective of biomarker and drug development.

## Data Availability

No datasets were generated or analyzed during the current study.

## References

[1] Fredriksson, L., Li, H., & Eriksson, U. (2004). The PDGF family: Four gene products form five dimeric isoforms. *Cytokine & Growth Factor Reviews,**15*(4), 197–204. 10.1016/j.cytogfr.2004.03.007 15207811 10.1016/j.cytogfr.2004.03.007

[2] Bonner, J. C. (2006). Platelet-derived growth factor. In G. J. Laurent & S. D. Shapiro (Eds.), *Encyclopedia of Respiratory Medicine* (pp. 343–347). Academic Press. 10.1016/B0-12-370879-6/00297-0

[3] Kazlauskas, A. (2017). PDGFs and their receptors. *Gene,**614*, 1–7. 10.1016/j.gene.2017.03.003 28267575 10.1016/j.gene.2017.03.003 PMC6728141

[4] Heldin, C.-H., Lennartsson, J., & Westermark, B. (2018). Involvement of platelet-derived growth factor ligands and receptors in tumorigenesis. *Journal of Internal Medicine,**283*(1), 16–44. 10.1111/joim.12690 28940884 10.1111/joim.12690

[5] Betsholtz, C., Johnsson, A., Heldin, C. H., Westermark, B., Lind, P., Urdea, M. S., … Mellor, A. L. (1986). cDNA sequence and chromosomal localization of human platelet-derived growth factor A-chain and its expression in tumour cell lines. *Nature*, *320*(6064), 695–699. 10.1038/320695a0 10.1038/320695a0 3754619

[6] Andrae, J., Gallini, R., & Betsholtz, C. (2008). Role of platelet-derived growth factors in physiology and medicine. *Genes & Development,**22*(10), 1276–1312. 10.1101/gad.1653708 18483217 10.1101/gad.1653708 PMC2732412

[7] Ostman, A., Andersson, M., Bäckström, G., & Heldin, C. H. (1993). Assignment of intrachain disulfide bonds in platelet-derived growth factor B-chain. *The Journal of Biological Chemistry,**268*(18), 13372–13377. 8514775

[8] Ostman, A., Andersson, M., Betsholtz, C., Westermark, B., & Heldin, C. H. (1991). Identification of a cell retention signal in the B-chain of platelet-derived growth factor and in the long splice version of the A-chain. *Cell Regulation,**2*(7), 503–512. 10.1091/mbc.2.7.503 1782212 10.1091/mbc.2.7.503 PMC361840

[9] Betsholtz, C., Rorsman, F., Westermark, B., Ostman, A., & Heldin, C. H. (1990). Analogous alternative splicing. *Nature,**344*(6264), 299. 10.1038/344299a0 2314468 10.1038/344299a0

[10] Claesson-Welsh, L., Eriksson, A., Westermark, B., & Heldin, C. H. (1989). cDNA cloning and expression of the human A-type platelet-derived growth factor (PDGF) receptor establishes structural similarity to the B-type PDGF receptor. *Proceedings of the National Academy of Sciences of the United States of America,**86*(13), 4917–4921. 10.1073/pnas.86.13.4917 2544881 10.1073/pnas.86.13.4917 PMC297526

[11] Heldin, C.-H., & Lennartsson, J. (2013). Structural and functional properties of platelet-derived growth factor and stem cell factor receptors. *Cold Spring Harbor Perspectives in Biology,**5*(8), a009100. 10.1101/cshperspect.a009100 23906712 10.1101/cshperspect.a009100 PMC3721287

[12] Miyazawa, K., Bäckström, G., Leppänen, O., Persson, C., Wernstedt, C., Hellman, U., … Ostman, A. (1998). Role of immunoglobulin-like domains 2–4 of the platelet-derived growth factor alpha-receptor in ligand-receptor complex assembly. *The Journal of Biological Chemistry*, *273*(39), 25495–25502. 10.1074/jbc.273.39.25495 10.1074/jbc.273.39.25495 9738020

[13] Fredriksson, L., Ehnman, M., Fieber, C., & Eriksson, U. (2005). Structural requirements for activation of latent platelet-derived growth factor CC by tissue plasminogen activator. *The Journal of Biological Chemistry,**280*(29), 26856–26862. 10.1074/jbc.M503388200 15911618 10.1074/jbc.M503388200

[14] Heldin, C. H., Ostman, A., & Rönnstrand, L. (1998). Signal transduction via platelet-derived growth factor receptors. *Biochimica Et Biophysica Acta,**1378*(1), F79-113. 10.1016/s0304-419x(98)00015-8 9739761 10.1016/s0304-419x(98)00015-8

[15] Basciani, S., Mariani, S., Spera, G., & Gnessi, L. (2010). Role of platelet-derived growth factors in the testis. *Endocrine Reviews,**31*(6), 916–939. 10.1210/er.2010-0004 20650860 10.1210/er.2010-0004

[16] Campaña, M. B., Perkins, M. R., McCabe, M. C., Neumann, A., Larson, E. D., & Fantauzzo, K. A. (2023). PDGFRα/β heterodimer activation negatively affects downstream ERK1/2 signaling and cellular proliferation. *bioRxiv*, 2023.12.27.573428. 10.1101/2023.12.27.573428 10.1038/s41467-025-59938-1 PMC12098797 40404618

[17] Ekman, S., Kallin, A., Engström, U., Heldin, C.-H., & Rönnstrand, L. (2002). SHP-2 is involved in heterodimer specific loss of phosphorylation of Tyr771 in the PDGF beta-receptor. *Oncogene,**21*(12), 1870–1875. 10.1038/sj.onc.1205210 11896619 10.1038/sj.onc.1205210

[18] Heldin, C.-H. (2013). Targeting the PDGF signaling pathway in tumor treatment. *Cell Communication and Signaling: CCS,**11*, 97. 10.1186/1478-811X-11-97 24359404 10.1186/1478-811X-11-97 PMC3878225

[19] Goh, L. K., & Sorkin, A. (2013). Endocytosis of receptor tyrosine kinases. *Cold Spring Harbor Perspectives in Biology*, *5*(5). 10.1101/cshperspect.a017459 10.1101/cshperspect.a017459 PMC3632065 23637288

[20] Levkowitz, G., Waterman, H., Ettenberg, S. A., Katz, M., Tsygankov, A. Y., Alroy, I., … Yarden, Y. (1999). Ubiquitin ligase activity and tyrosine phosphorylation underlie suppression of growth factor signaling by c-Cbl/Sli-1. *Molecular Cell*, *4*(6), 1029–1040. 10.1016/s1097-2765(00)80231-2 10.1016/s1097-2765(00)80231-2 10635327

[21] Dagnell, M., Frijhoff, J., Pader, I., Augsten, M., Boivin, B., Xu, J., … Östman, A. (2013). Selective activation of oxidized PTP1B by the thioredoxin system modulates PDGF-β receptor tyrosine kinase signaling. *Proceedings of the National Academy of Sciences of the United States of America*, *110*(33), 13398–13403. 10.1073/pnas.1302891110 10.1073/pnas.1302891110 PMC3746926 23901112

[22] Persson, C., Sävenhed, C., Bourdeau, A., Tremblay, M. L., Markova, B., Böhmer, F. D., … Hellberg, C. (2004). Site-selective regulation of platelet-derived growth factor beta receptor tyrosine phosphorylation by T-cell protein tyrosine phosphatase. *Molecular and Cellular Biology*, *24*(5), 2190–2201. 10.1128/MCB.24.5.2190-2201.2004 10.1128/MCB.24.5.2190-2201.2004 PMC350555 14966296

[23] Fantauzzo, K. A., & Soriano, P. (2016). PDGFRβ regulates craniofacial development through homodimers and functional heterodimers with PDGFRα. *Genes & Development,**30*(21), 2443–2458. 10.1101/gad.288746.116 27856617 10.1101/gad.288746.116 PMC5131783

[24] Ding, H., Wu, X., Boström, H., Kim, I., Wong, N., Tsoi, B., … Nagy, A. (2004). A specific requirement for PDGF-C in palate formation and PDGFR-alpha signaling. *Nature Genetics*, *36*(10), 1111–1116. 10.1038/ng1415 10.1038/ng1415 15361870

[25] Soriano, P. (1997). The PDGF alpha receptor is required for neural crest cell development and for normal patterning of the somites. *Development,**124*(14), 2691–2700. 9226440 10.1242/dev.124.14.2691

[26] Lindahl, P., Karlsson, L., Hellström, M., Gebre-Medhin, S., Willetts, K., Heath, J. K., & Betsholtz, C. (1997). Alveogenesis failure in PDGF-A-deficient mice is coupled to lack of distal spreading of alveolar smooth muscle cell progenitors during lung development. *Development,**124*(20), 3943–3953. 9374392 10.1242/dev.124.20.3943

[27] Boström, H., Willetts, K., Pekny, M., Levéen, P., Lindahl, P., Hedstrand, H., … Betsholtz, C. (1996). PDGF-A signaling is a critical event in lung alveolar myofibroblast development and alveogenesis. *Cell*, *85*(6), 863–873. 10.1016/s0092-8674(00)81270-2 10.1016/s0092-8674(00)81270-2 8681381

[28] Karlsson, L., Lindahl, P., Heath, J. K., & Betsholtz, C. (2000). Abnormal gastrointestinal development in PDGF-A and PDGFR-(alpha) deficient mice implicates a novel mesenchymal structure with putative instructive properties in villus morphogenesis. *Development,**127*(16), 3457–3466. 10903171 10.1242/dev.127.16.3457

[29] Greicius, G., Kabiri, Z., Sigmundsson, K., Liang, C., Bunte, R., Singh, M. K., & Virshup, D. M. (2018). PDGFRα + pericryptal stromal cells are the critical source of Wnts and RSPO3 for murine intestinal stem cells in vivo. *Proceedings of the National Academy of Sciences of the United States of America,**115*(14), E3173–E3181. 10.1073/pnas.1713510115 29559533 10.1073/pnas.1713510115 PMC5889626

[30] Kinchen, J., Chen, H. H., Parikh, K., Antanaviciute, A., Jagielowicz, M., Fawkner-Corbett, D., … Simmons, A. (2018). Structural remodeling of the human colonic mesenchyme in inflammatory bowel disease. *Cell*, *175*(2), 372–386.e17. 10.1016/j.cell.2018.08.067 10.1016/j.cell.2018.08.067 PMC6176871 30270042

[31] Karlsson, L., Bondjers, C., & Betsholtz, C. (1999). Roles for PDGF-A and sonic hedgehog in development of mesenchymal components of the hair follicle. *Development,**126*(12), 2611–2621. 10331973 10.1242/dev.126.12.2611

[32] Shin, S., Pang, Y., Park, J., Liu, L., Lukas, B. E., Kim, S. H., … Jiang, Y. (2020). Dynamic control of adipose tissue development and adult tissue homeostasis by platelet-derived growth factor receptor alpha. *eLife*, *9*, e56189. 10.7554/eLife.56189 10.7554/eLife.56189 PMC7338051 32553115

[33] Wosczyna, M. N., Konishi, C. T., Perez Carbajal, E. E., Wang, T. T., Walsh, R. A., Gan, Q., … Rando, T. A. (2019). Mesenchymal stromal cells are required for regeneration and homeostatic maintenance of skeletal muscle. *Cell Reports*, *27*(7), 2029–2035.e5. 10.1016/j.celrep.2019.04.074 10.1016/j.celrep.2019.04.074 PMC7034941 31091443

[34] Calver, A. R., Hall, A. C., Yu, W. P., Walsh, F. S., Heath, J. K., Betsholtz, C., & Richardson, W. D. (1998). Oligodendrocyte population dynamics and the role of PDGF in vivo. *Neuron,**20*(5), 869–882. 10.1016/s0896-6273(00)80469-9 9620692 10.1016/s0896-6273(00)80469-9

[35] Fruttiger, M., Karlsson, L., Hall, A. C., Abramsson, A., Calver, A. R., Boström, H., … Richardson, W. D. (1999). Defective oligodendrocyte development and severe hypomyelination in PDGF-A knockout mice. *Development*, *126*(3), 457–467. 10.1242/dev.126.3.457 9876175

[36] Bonner, J. C., Osornio-Vargas, A. R., Badgett, A., & Brody, A. R. (1991). Differential proliferation of rat lung fibroblasts induced by the platelet-derived growth factor-AA, -AB, and -BB isoforms secreted by rat alveolar macrophages. *American Journal of Respiratory Cell and Molecular Biology,**5*(6), 539–547. 10.1165/ajrcmb/5.6.539 1958381 10.1165/ajrcmb/5.6.539

[37] Abdollahi, A., Li, M., Ping, G., Plathow, C., Domhan, S., Kiessling, F., … Huber, P. E. (2005). Inhibition of platelet-derived growth factor signaling attenuates pulmonary fibrosis. *The Journal of Experimental Medicine*, *201*(6), 925–935. 10.1084/jem.20041393 10.1084/jem.20041393 PMC2213091 15781583

[38] Takemura, H., Suzuki, H., Fujisawa, H., Yuhara, T., Akama, T., Yamane, K., & Kashiwagi, H. (1998). Enhanced interleukin 6 production by cultured fibroblasts from patients with systemic sclerosis in response to platelet derived growth factor. *The Journal of Rheumatology,**25*(8), 1534–1539. 9712097

[39] Iwayama, T., Steele, C., Yao, L., Dozmorov, M. G., Karamichos, D., Wren, J. D., & Olson, L. E. (2015). PDGFRα signaling drives adipose tissue fibrosis by targeting progenitor cell plasticity. *Genes & Development,**29*(11), 1106–1119. 10.1101/gad.260554.115 26019175 10.1101/gad.260554.115 PMC4470280

[40] Marcelin, G., Ferreira, A., Liu, Y., Atlan, M., Aron-Wisnewsky, J., Pelloux, V., … Clément, K. (2017). A PDGFRα-mediated switch toward CD9high adipocyte progenitors controls obesity-induced adipose tissue fibrosis. *Cell Metabolism*, *25*(3), 673–685. 10.1016/j.cmet.2017.01.010 10.1016/j.cmet.2017.01.010 28215843

[41] Betsholtz, C. (2004). Insight into the physiological functions of PDGF through genetic studies in mice. *Cytokine & Growth Factor Reviews,**15*(4), 215–228. 10.1016/j.cytogfr.2004.03.005 15207813 10.1016/j.cytogfr.2004.03.005

[42] Hoch, R. V., & Soriano, P. (2003). Roles of PDGF in animal development. *Development,**130*(20), 4769–4784. 10.1242/dev.00721 12952899 10.1242/dev.00721

[43] Lindahl, P., Johansson, B. R., Levéen, P., & Betsholtz, C. (1997). Pericyte loss and microaneurysm formation in PDGF-B-deficient mice. *Science,**277*(5323), 242–245. 10.1126/science.277.5323.242 9211853 10.1126/science.277.5323.242

[44] Hellström, M., Gerhardt, H., Kalén, M., Li, X., Eriksson, U., Wolburg, H., & Betsholtz, C. (2001). Lack of pericytes leads to endothelial hyperplasia and abnormal vascular morphogenesis. *The Journal of Cell Biology,**153*(3), 543–554. 11331305 10.1083/jcb.153.3.543 PMC2190573

[45] Bjarnegård, M., Enge, M., Norlin, J., Gustafsdottir, S., Fredriksson, S., Abramsson, A., … Betsholtz, C. (2004). Endothelium-specific ablation of PDGFB leads to pericyte loss and glomerular, cardiac and placental abnormalities. *Development*, *131*(8), 1847–1857. 10.1242/dev.01080 10.1242/dev.01080 15084468

[46] Soriano, P. (1994). Abnormal kidney development and hematological disorders in PDGF beta-receptor mutant mice. *Genes & Development,**8*(16), 1888–1896. 10.1101/gad.8.16.1888 7958864 10.1101/gad.8.16.1888

[47] Levéen, P., Pekny, M., Gebre-Medhin, S., Swolin, B., Larsson, E., & Betsholtz, C. (1994). Mice deficient for PDGF B show renal, cardiovascular, and hematological abnormalities. *Genes & Development,**8*(16), 1875–1887. 10.1101/gad.8.16.1875 7958863 10.1101/gad.8.16.1875

[48] Lindblom, P., Gerhardt, H., Liebner, S., Abramsson, A., Enge, M., Hellstrom, M., … Betsholtz, C. (2003). Endothelial PDGF-B retention is required for proper investment of pericytes in the microvessel wall. *Genes & Development*, *17*(15), 1835–1840. 10.1101/gad.266803 10.1101/gad.266803 PMC196228 12897053

[49] Betsholtz, C., & Keller, A. (2014). PDGF, pericytes and the pathogenesis of idiopathic basal ganglia calcification (IBGC). *Brain Pathology,**24*(4), 387–395. 10.1111/bpa.12158 24946076 10.1111/bpa.12158 PMC8029277

[50] He, C., Medley, S. C., Hu, T., Hinsdale, M. E., Lupu, F., Virmani, R., & Olson, L. E. (2015). PDGFRβ signalling regulates local inflammation and synergizes with hypercholesterolaemia to promote atherosclerosis. *Nature Communications,**6*(1), 7770. 10.1038/ncomms8770 26183159 10.1038/ncomms8770 PMC4507293

[51] Kozaki, K., Kaminski, W. E., Tang, J., Hollenbach, S., Lindahl, P., Sullivan, C., … Raines, E. W. (2002). Blockade of platelet-derived growth factor or its receptors transiently delays but does not prevent fibrous cap formation in ApoE null mice. *The American Journal of Pathology*, *161*(4), 1395–1407. 10.1016/S0002-9440(10)64415-X 10.1016/S0002-9440(10)64415-X PMC1867295 12368212

[52] Bilder, G., Wentz, T., Leadley, R., Amin, D., Byan, L., O’Conner, B., … Dunwiddie, C. (1999). Restenosis following angioplasty in the swine coronary artery is inhibited by an orally active PDGF-receptor tyrosine kinase inhibitor, RPR101511A. *Circulation*, *99*(25), 3292–3299. 10.1161/01.cir.99.25.3292 10.1161/01.cir.99.25.3292 10385505

[53] Nakamura, K., Arimura, K., Nishimura, A., Tachibana, M., Yoshikawa, Y., Makihara, N., … Ago, T. (2016). Possible involvement of basic FGF in the upregulation of PDGFRβ in pericytes after ischemic stroke. *Brain Research*, *1630*, 98–108. 10.1016/j.brainres.2015.11.003 10.1016/j.brainres.2015.11.003 26569132

[54] Kanazawa, S., Miyake, T., Kakinuma, T., Tanemoto, K., Tsunoda, T., & Kikuchi, K. (2005). The expression of platelet-derived growth factor and connective tissue growth factor in different types of abdominal aortic aneurysms. *The Journal of Cardiovascular Surgery,**46*(3), 271–278. 15956925

[55] Vorkapic, E., Dugic, E., Vikingsson, S., Roy, J., Mäyränpää, M. I., Eriksson, P., & Wågsäter, D. (2016). Imatinib treatment attenuates growth and inflammation of angiotensin II induced abdominal aortic aneurysm. *Atherosclerosis,**249*, 101–109. 10.1016/j.atherosclerosis.2016.04.006 27085160 10.1016/j.atherosclerosis.2016.04.006

[56] Tannenberg, P., Chang, Y.-T., Muhl, L., Laviña, B., Gladh, H., Genové, G., … Tran-Lundmark, K. (2018). Extracellular retention of PDGF-B directs vascular remodeling in mouse hypoxia-induced pulmonary hypertension. *American Journal of Physiology. Lung Cellular and Molecular Physiology*, *314*(4), L593–L605. 10.1152/ajplung.00054.2017 10.1152/ajplung.00054.2017 29212800

[57] Schermuly, R. T., Dony, E., Ghofrani, H. A., Pullamsetti, S., Savai, R., Roth, M., … Grimminger, F. (2005). Reversal of experimental pulmonary hypertension by PDGF inhibition. *The Journal of Clinical Investigation*, *115*(10), 2811–2821. 10.1172/JCI24838 10.1172/JCI24838 PMC1236676 16200212

[58] Forsberg, K., Valyi-Nagy, I., Heldin, C. H., Herlyn, M., & Westermark, B. (1993). Platelet-derived growth factor (PDGF) in oncogenesis: Development of a vascular connective tissue stroma in xenotransplanted human melanoma producing PDGF-BB. *Proceedings of the National Academy of Sciences of the United States of America,**90*(2), 393–397. 10.1073/pnas.90.2.393 8380638 10.1073/pnas.90.2.393 PMC45668

[59] Skobe, M., & Fusenig, N. E. (1998). Tumorigenic conversion of immortal human keratinocytes through stromal cell activation. *Proceedings of the National Academy of Sciences of the United States of America,**95*(3), 1050–1055. 10.1073/pnas.95.3.1050 9448283 10.1073/pnas.95.3.1050 PMC18668

[60] Tejada, M. L., Yu, L., Dong, J., Jung, K., Meng, G., Peale, F. V., … Ferrara, N. (2006). Tumor-driven paracrine platelet-derived growth factor receptor alpha signaling is a key determinant of stromal cell recruitment in a model of human lung carcinoma. *Clinical Cancer Research: An Official Journal of the American Association for Cancer Research*, *12*(9), 2676–2688. 10.1158/1078-0432.CCR-05-1770 10.1158/1078-0432.CCR-05-1770 16675559

[61] Campbell, J. S., Hughes, S. D., Gilbertson, D. G., Palmer, T. E., Holdren, M. S., Haran, A. C., … Fausto, N. (2005). Platelet-derived growth factor C induces liver fibrosis, steatosis, and hepatocellular carcinoma. *Proceedings of the National Academy of Sciences of the United States of America*, *102*(9), 3389–3394. 10.1073/pnas.0409722102 10.1073/pnas.0409722102 PMC552940 15728360

[62] Anderberg, C., Li, H., Fredriksson, L., Andrae, J., Betsholtz, C., Li, X., … Pietras, K. (2009). Paracrine signaling by platelet-derived growth factor-CC promotes tumor growth by recruitment of cancer-associated fibroblasts. *Cancer Research*, *69*(1), 369–378. 10.1158/0008-5472.CAN-08-2724 10.1158/0008-5472.CAN-08-2724 PMC2613547 19118022

[63] Shao, Z. M., Nguyen, M., & Barsky, S. H. (2000). Human breast carcinoma desmoplasia is PDGF initiated. *Oncogene,**19*(38), 4337–4345. 10.1038/sj.onc.1203785 10980609 10.1038/sj.onc.1203785

[64] Pietras, K., Pahler, J., Bergers, G., & Hanahan, D. (2008). Functions of paracrine PDGF signaling in the proangiogenic tumor stroma revealed by pharmacological targeting. *PLoS Medicine*, *5*(1). 10.1371/journal.pmed.0050019 10.1371/journal.pmed.0050019 PMC2214790 18232728

[65] Orimo, A., Gupta, P. B., Sgroi, D. C., Arenzana-Seisdedos, F., Delaunay, T., Naeem, R., … Weinberg, R. A. (2005). Stromal fibroblasts present in invasive human breast carcinomas promote tumor growth and angiogenesis through elevated SDF-1/CXCL12 secretion. *Cell*, *121*(3), 335–348. 10.1016/j.cell.2005.02.034 10.1016/j.cell.2005.02.034 15882617

[66] Lederle, W., Stark, H.-J., Skobe, M., Fusenig, N. E., & Mueller, M. M. (2006). Platelet-derived growth factor-BB controls epithelial tumor phenotype by differential growth factor regulation in stromal cells. *The American Journal of Pathology,**169*(5), 1767–1783. 10.2353/ajpath.2006.060120 17071599 10.2353/ajpath.2006.060120 PMC1780216

[67] Strell, C., Paulsson, J., Jin, S.-B., Tobin, N. P., Mezheyeuski, A., Roswall, P., … Östman, A. (2019). Impact of epithelial-stromal interactions on peritumoral fibroblasts in ductal carcinoma in situ. *Journal of the National Cancer Institute*, *111*(9), 983–995. 10.1093/jnci/djy234 10.1093/jnci/djy234 PMC6748730 30816935

[68] Raz, Y., Cohen, N., Shani, O., Bell, R. E., Novitskiy, S. V., Abramovitz, L., … Erez, N. (2018). Bone marrow-derived fibroblasts are a functionally distinct stromal cell population in breast cancer. *The Journal of Experimental Medicine*, *215*(12), 3075–3093. 10.1084/jem.20180818 10.1084/jem.20180818 PMC6279405 30470719

[69] Bartoschek, M., Oskolkov, N., Bocci, M., Lövrot, J., Larsson, C., Sommarin, M., … Pietras, K. (2018). Spatially and functionally distinct subclasses of breast cancer-associated fibroblasts revealed by single cell RNA sequencing. *Nature Communications*, *9*(1), 5150. 10.1038/s41467-018-07582-3 10.1038/s41467-018-07582-3 PMC6279758 30514914

[70] Abramsson, A., Lindblom, P., & Betsholtz, C. (2003). Endothelial and nonendothelial sources of PDGF-B regulate pericyte recruitment and influence vascular pattern formation in tumors. *The Journal of Clinical Investigation,**112*(8), 1142–1151. 10.1172/JCI18549 14561699 10.1172/JCI18549 PMC213487

[71] Furuhashi, M., Sjöblom, T., Abramsson, A., Ellingsen, J., Micke, P., Li, H., … Ostman, A. (2004). Platelet-derived growth factor production by B16 melanoma cells leads to increased pericyte abundance in tumors and an associated increase in tumor growth rate. *Cancer Research*, *64*(8), 2725–2733. 10.1158/0008-5472.can-03-1489 10.1158/0008-5472.can-03-1489 15087386

[72] Awazu, Y., Mizutani, A., Nagase, Y., Tsuchiya, S., Nakamura, K., Kakoi, Y., … Hori, A. (2013). Anti-angiogenic and anti-tumor effects of TAK-593, a potent and selective inhibitor of vascular endothelial growth factor and platelet-derived growth factor receptor tyrosine kinase. *Cancer Science*, *104*(4), 486–494. 10.1111/cas.12101 10.1111/cas.12101 PMC7657107 23305239

[73] Hasumi, Y., Kłosowska-Wardega, A., Furuhashi, M., Ostman, A., Heldin, C.-H., & Hellberg, C. (2007). Identification of a subset of pericytes that respond to combination therapy targeting PDGF and VEGF signaling. *International Journal of Cancer,**121*(12), 2606–2614. 10.1002/ijc.22999 17691110 10.1002/ijc.22999

[74] Erber, R., Thurnher, A., Katsen, A. D., Groth, G., Kerger, H., Hammes, H.-P., … Vajkoczy, P. (2004). Combined inhibition of VEGF and PDGF signaling enforces tumor vessel regression by interfering with pericyte-mediated endothelial cell survival mechanisms. *FASEB journal: official publication of the Federation of American Societies for Experimental Biology*, *18*(2), 338–340. 10.1096/fj.03-0271fje 10.1096/fj.03-0271fje 14657001

[75] Thijssen, V. L., Paulis, Y. W., Nowak-Sliwinska, P., Deumelandt, K. L., Hosaka, K., Soetekouw, P. M., … Griffioen, A. W. (2018). Targeting PDGF-mediated recruitment of pericytes blocks vascular mimicry and tumor growth. *The Journal of Pathology*, *246*(4), 447–458. 10.1002/path.5152 10.1002/path.5152 PMC6587443 30101525

[76] Shinagawa, K., Kitadai, Y., Tanaka, M., Sumida, T., Onoyama, M., Ohnishi, M., … Chayama, K. (2013). Stroma-directed imatinib therapy impairs the tumor-promoting effect of bone marrow-derived mesenchymal stem cells in an orthotopic transplantation model of colon cancer. *International Journal of Cancer*, *132*(4), 813–823. 10.1002/ijc.27735 10.1002/ijc.27735 22821812

[77] Peña, C., Céspedes, M. V., Lindh, M. B., Kiflemariam, S., Mezheyeuski, A., Edqvist, P.-H., … Ostman, A. (2013). STC1 expression by cancer-associated fibroblasts drives metastasis of colorectal cancer. *Cancer Research*, *73*(4), 1287–1297. 10.1158/0008-5472.CAN-12-1875 10.1158/0008-5472.CAN-12-1875 23243022

[78] Liu, C., Zhang, Y., Lim, S., Hosaka, K., Yang, Y., Pavlova, T., … Cao, Y. (2017). A zebrafish model discovers a novel mechanism of stromal fibroblast-mediated cancer metastasis. *Clinical Cancer Research*, *23*(16), 4769–4779. 10.1158/1078-0432.CCR-17-0101 10.1158/1078-0432.CCR-17-0101 28420724

[79] Cooke, V. G., LeBleu, V. S., Keskin, D., Khan, Z., O’Connell, J. T., Teng, Y., … Kalluri, R. (2012). Pericyte depletion results in hypoxia-associated epithelial-to-mesenchymal transition and metastasis mediated by met signaling pathway. *Cancer Cell*, *21*(1), 66–81. 10.1016/j.ccr.2011.11.024 10.1016/j.ccr.2011.11.024 PMC3999522 22264789

[80] Keskin, D., Kim, J., Cooke, V. G., Wu, C.-C., Sugimoto, H., Gu, C., … LeBleu, V. S. (2015). Targeting vascular pericytes in hypoxic tumors increases lung metastasis via angiopoietin-2. *Cell Reports*, *10*(7), 1066–1081. 10.1016/j.celrep.2015.01.035 10.1016/j.celrep.2015.01.035 PMC4342328 25704811

[81] Hosaka, K., Yang, Y., Seki, T., Nakamura, M., Andersson, P., Rouhi, P., … Cao, Y. (2013). Tumour PDGF-BB expression levels determine dual effects of anti-PDGF drugs on vascular remodelling and metastasis. *Nature Communications*, *4*, 2129. 10.1038/ncomms3129 10.1038/ncomms3129 23831851

[82] Thies, K. A., Hammer, A. M., Hildreth, B. E., Steck, S. A., Spehar, J. M., Kladney, R. D., … Sizemore, G. M. (2021). Stromal platelet-derived growth factor receptor-β signaling promotes breast cancer metastasis in the brain. *Cancer Research*, *81*(3), 606–618. 10.1158/0008-5472.CAN-19-3731 10.1158/0008-5472.CAN-19-3731 PMC7581545 32327406

[83] Wyss, C. B., Duffey, N., Peyvandi, S., Barras, D., Martinez Usatorre, A., Coquoz, O., … Rüegg, C. (2021). Gain of HIF1 activity and loss of miRNA let-7d promote breast cancer metastasis to the brain via the PDGF/PDGFR axis. *Cancer Research*, *81*(3), 594–605. 10.1158/0008-5472.CAN-19-3560 10.1158/0008-5472.CAN-19-3560 33526470

[84] Primac, I., Maquoi, E., Blacher, S., Heljasvaara, R., Van Deun, J., Smeland, H. Y., … Noel, A. (2019). Stromal integrin α11 regulates PDGFR-β signaling and promotes breast cancer progression. *The Journal of Clinical Investigation*, *129*(11), 4609–4628. 10.1172/JCI125890 10.1172/JCI125890 PMC6819106 31287804

[85] Zhang, Y., Cedervall, J., Hamidi, A., Herre, M., Viitaniemi, K., D’Amico, G., … Olsson, A.-K. (2020). Platelet-specific PDGFB ablation impairs tumor vessel integrity and promotes metastasis. *Cancer Research*, *80*(16), 3345–3358. 10.1158/0008-5472.CAN-19-3533 10.1158/0008-5472.CAN-19-3533 32586981

[86] Wu, R., Gandhi, S., Tokumaru, Y., Asaoka, M., Oshi, M., Yan, L., … Takabe, K. (2022). Intratumoral PDGFB gene predominantly expressed in endothelial cells is associated with angiogenesis and lymphangiogenesis, but not with metastasis in breast cancer. *Breast Cancer Research and Treatment*, *195*(1), 17–31. 10.1007/s10549-022-06661-w 10.1007/s10549-022-06661-w PMC12118772 35793004

[87] Georgoudaki, A.-M., Prokopec, K. E., Boura, V. F., Hellqvist, E., Sohn, S., Östling, J., … Karlsson, M. C. I. (2016). Reprogramming tumor-associated macrophages by antibody targeting inhibits cancer progression and metastasis. *Cell Reports*, *15*(9), 2000–2011. 10.1016/j.celrep.2016.04.084 10.1016/j.celrep.2016.04.084 27210762

[88] Hsu, Y.-L., Yen, M.-C., Chang, W.-A., Tsai, P.-H., Pan, Y.-C., Liao, S.-H., & Kuo, P.-L. (2019). CXCL17-derived CD11b+Gr-1+ myeloid-derived suppressor cells contribute to lung metastasis of breast cancer through platelet-derived growth factor-BB. *Breast Cancer Research: BCR,**21*(1), 23. 10.1186/s13058-019-1114-3 30755260 10.1186/s13058-019-1114-3 PMC6373011

[89] Reed, R. K., & Rubin, K. (2010). Transcapillary exchange: Role and importance of the interstitial fluid pressure and the extracellular matrix. *Cardiovascular Research,**87*(2), 211–217. 10.1093/cvr/cvq143 20472565 10.1093/cvr/cvq143

[90] Baranowska-Kortylewicz, J., Abe, M., Pietras, K., Kortylewicz, Z. P., Kurizaki, T., Nearman, J., … Ostman, A. (2005). Effect of platelet-derived growth factor receptor-beta inhibition with STI571 on radioimmunotherapy. *Cancer Research*, *65*(17), 7824–7831. 10.1158/0008-5472.CAN-04-3991 10.1158/0008-5472.CAN-04-3991 PMC1363769 16140951

[91] Pietras, K., Stumm, M., Hubert, M., Buchdunger, E., Rubin, K., Heldin, C.-H., … Ostman, A. (2003). STI571 enhances the therapeutic index of epothilone B by a tumor-selective increase of drug uptake. *Clinical Cancer Research: An Official Journal of the American Association for Cancer Research*, *9*(10 Pt 1), 3779–3787. 14506171

[92] Pietras, K., Rubin, K., Sjöblom, T., Buchdunger, E., Sjöquist, M., Heldin, C.-H., & Ostman, A. (2002). Inhibition of PDGF receptor signaling in tumor stroma enhances antitumor effect of chemotherapy. *Cancer Research,**62*(19), 5476–5484. 12359756

[93] Pietras, K., Ostman, A., Sjöquist, M., Buchdunger, E., Reed, R. K., Heldin, C. H., & Rubin, K. (2001). Inhibition of platelet-derived growth factor receptors reduces interstitial hypertension and increases transcapillary transport in tumors. *Cancer Research,**61*(7), 2929–2934. 11306470

[94] Roswall, P., Bocci, M., Bartoschek, M., Li, H., Kristiansen, G., Jansson, S., … Pietras, K. (2018). Microenvironmental control of breast cancer subtype elicited through paracrine platelet-derived growth factor-CC signaling. *Nature Medicine*, *24*(4), 463–473. 10.1038/nm.4494 10.1038/nm.4494 PMC5896729 29529015

[95] Paulsson, J., Sjöblom, T., Micke, P., Pontén, F., Landberg, G., Heldin, C.-H., … Östman, A. (2009). Prognostic significance of stromal platelet-derived growth factor β-receptor expression in human breast cancer. *The American Journal of Pathology*, *175*(1), 334–341. 10.2353/ajpath.2009.081030 10.2353/ajpath.2009.081030 PMC2708819 19498003

[96] Frings, O., Augsten, M., Tobin, N. P., Carlson, J., Paulsson, J., Pena, C., … Sonnhammer, E. L. L. (2013). Prognostic significance in breast cancer of a gene signature capturing stromal PDGF signaling. *The American Journal of Pathology*, *182*(6), 2037–2047. 10.1016/j.ajpath.2013.02.018 10.1016/j.ajpath.2013.02.018 23583284

[97] Strell, C., Stenmark Tullberg, A., Jetne Edelmann, R., Akslen, L. A., Malmström, P., Fernö, M., … Karlsson, P. (2021). Prognostic and predictive impact of stroma cells defined by PDGFRb expression in early breast cancer: Results from the randomized SweBCG91RT trial. *Breast Cancer Research and Treatment*, *187*(1), 45–55. 10.1007/s10549-021-06136-4 10.1007/s10549-021-06136-4 PMC8062362 33661437

[98] Corvigno, S., Wisman, G. B. A., Mezheyeuski, A., van der Zee, A. G. J., Nijman, H. W., Åvall-Lundqvist, E., … Dahlstrand, H. (2016). Markers of fibroblast-rich tumor stroma and perivascular cells in serous ovarian cancer: Inter- and intra-patient heterogeneity and impact on survival. *Oncotarget*, *7*(14), 18573–18584. 10.18632/oncotarget.7613 10.18632/oncotarget.7613 PMC4951310 26918345

[99] Sun, W.-Y., Jung, W.-H., & Koo, J. S. (2016). Expression of cancer-associated fibroblast-related proteins in thyroid papillary carcinoma. *Tumour Biology: The Journal of the International Society for Oncodevelopmental Biology and Medicine,**37*(6), 8197–8207. 10.1007/s13277-015-4684-4 26715280 10.1007/s13277-015-4684-4

[100] Hägglöf, C., Hammarsten, P., Josefsson, A., Stattin, P., Paulsson, J., Bergh, A., & Östman, A. (2010). Stromal PDGFRβ expression in prostate tumors and non-malignant prostate tissue predicts prostate cancer survival. *PLoS ONE*, *5*(5). 10.1371/journal.pone.0010747 10.1371/journal.pone.0010747 PMC2873980 20505768

[101] Nordby, Y., Richardsen, E., Rakaee, M., Ness, N., Donnem, T., Patel, H. R. H., … Andersen, S. (2017). High expression of PDGFR-β in prostate cancer stroma is independently associated with clinical and biochemical prostate cancer recurrence. *Scientific Reports*, *7*. 10.1038/srep43378 10.1038/srep43378 PMC5324133 28233816

[102] Frödin, M., Mezheyeuski, A., Corvigno, S., Harmenberg, U., Sandström, P., Egevad, L., … Östman, A. (2017). Perivascular PDGFR- β is an independent marker for prognosis in renal cell carcinoma. *British Journal of Cancer*, *116*(2), 195–201. 10.1038/bjc.2016.407 10.1038/bjc.2016.407 PMC5243993 27931046

[103] Ehnman, M., Missiaglia, E., Folestad, E., Selfe, J., Strell, C., Thway, K., … Eriksson, U. (2013). Distinct effects of ligand-induced PDGFRα and PDGFRβ signaling in the human rhabdomyosarcoma tumor cell and stroma cell compartments. *Cancer Research*, *73*(7), 2139–2149. 10.1158/0008-5472.CAN-12-1646 10.1158/0008-5472.CAN-12-1646 PMC3672973 23338608

[104] Kilvaer, T. K., Rakaee, M., Hellevik, T., Vik, J., Petris, L. D., Donnem, T., … Martinez-Zubiaurre, I. (2019). Differential prognostic impact of platelet-derived growth factor receptor expression in NSCLC. *Scientific Reports*, *9*(1), 10163. 10.1038/s41598-019-46510-3 10.1038/s41598-019-46510-3 PMC6629689 31308421

[105] Pellinen, T., Paavolainen, L., Martín-Bernabé, A., Papatella Araujo, R., Strell, C., Mezheyeuski, A., … Östman, A. (2022). Fibroblast subsets in non-small cell lung cancer: Associations with survival, mutations, and immune features. *Journal of the National Cancer Institute*, djac178. 10.1093/jnci/djac178 10.1093/jnci/djac178 36083003

[106] Lin, L.-H., Lin, J.-S., Yang, C.-C., Cheng, H.-W., Chang, K.-W., & Liu, C.-J. (2020). Overexpression of platelet-derived growth factor and its receptor are correlated with oral tumorigenesis and poor prognosis in oral squamous cell carcinoma. *International Journal of Molecular Sciences,**21*(7), E2360. 10.3390/ijms21072360 10.3390/ijms21072360 PMC7177415 32235327

[107] Han, N., Zhang, Y.-Y., Zhang, Z.-M., Zhang, F., Zeng, T.-Y., Zhang, Y.-B., & Zhao, W.-C. (2021). High expression of PDGFA predicts poor prognosis of esophageal squamous cell carcinoma. *Medicine,**100*(20), e25932. 10.1097/MD.0000000000025932 34011067 10.1097/MD.0000000000025932 PMC8137088

[108] Paulsson, J., Rydén, L., Strell, C., Frings, O., Tobin, N. P., Fornander, T., … Östman, A. (2016). High expression of stromal PDGFRβ is associated with reduced benefit of tamoxifen in breast cancer. *The Journal of Pathology: Clinical Research*, *3*(1), 38–43. 10.1002/cjp2.56 10.1002/cjp2.56 PMC5259559 28138400

[109] Strell, C., Folkvaljon, D., Holmberg, E., Schiza, A., Thurfjell, V., Karlsson, P., … Östman, A. (2021). High PDGFRb expression predicts resistance to radiotherapy in DCIS within the SweDCIS randomized trial. *Clinical Cancer Research: An Official Journal of the American Association for Cancer Research*. 10.1158/1078-0432.CCR-20-4300 10.1158/1078-0432.CCR-20-4300 33952629

[110] Jansson, S., Aaltonen, K., Bendahl, P.-O., Falck, A.-K., Karlsson, M., Pietras, K., & Rydén, L. (2018). The PDGF pathway in breast cancer is linked to tumour aggressiveness, triple-negative subtype and early recurrence. *Breast Cancer Research and Treatment,**169*(2), 231–241. 10.1007/s10549-018-4664-7 29380207 10.1007/s10549-018-4664-7 PMC5945746

[111] Kodama, M., Kitadai, Y., Sumida, T., Ohnishi, M., Ohara, E., Tanaka, M., … Chayama, K. (2010). Expression of platelet-derived growth factor (PDGF)-B and PDGF-receptor β is associated with lymphatic metastasis in human gastric carcinoma. *Cancer Science*, *101*(9), 1984–1989. 10.1111/j.1349-7006.2010.01639.x 10.1111/j.1349-7006.2010.01639.x PMC11159455 20624165

[112] Kurahara, H., Maemura, K., Mataki, Y., Sakoda, M., Shinchi, H., & Natsugoe, S. (2016). Impact of p53 and PDGFR-β expression on metastasis and prognosis of patients with pancreatic cancer. *World Journal of Surgery,**40*(8), 1977–1984. 10.1007/s00268-016-3477-2 26940582 10.1007/s00268-016-3477-2

[113] Kitadai, Y., Sasaki, T., Kuwai, T., Nakamura, T., Bucana, C. D., Hamilton, S. R., & Fidler, I. J. (2006). Expression of activated platelet-derived growth factor receptor in stromal cells of human colon carcinomas is associated with metastatic potential. *International Journal of Cancer,**119*(11), 2567–2574. 10.1002/ijc.22229 16988946 10.1002/ijc.22229

[114] Yuzawa, S., Kano, M. R., Einama, T., & Nishihara, H. (2012). PDGFRβ expression in tumor stroma of pancreatic adenocarcinoma as a reliable prognostic marker. *Medical Oncology,**29*(4), 2824–2830. 10.1007/s12032-012-0193-0 22403002 10.1007/s12032-012-0193-0

[115] do Valle, I. B., Oliveira, S. R., da Silva, J. M., Peterle, G. T., Có, A. C. G., Sousa-Neto, S. S., … Silva, T. A. (2023). The participation of tumor residing pericytes in oral squamous cell carcinoma.*Scientific Reports*, *13*(1), 5460. 10.1038/s41598-023-32528-1 10.1038/s41598-023-32528-1 PMC10073133 37015965

[116] Colon-Echevarria, C. B., Ortiz, T., Maldonado, L., Hidalgo-Vargas, M. J., Pérez-Morales, J., Aquino-Acevedo, A. N., … Armaiz-Pena, G. N. (2020). Zoledronic acid abrogates restraint stress-induced macrophage infiltration, PDGF-AA expression, and ovarian cancer growth. *Cancers*, *12*(9), E2671. 10.3390/cancers12092671 10.3390/cancers12092671 PMC7563308 32962103

[117] Brahmi, M., Lesluyes, T., Dufresne, A., Toulmonde, M., Italiano, A., Mir, O., … Chibon, F. (2021). Expression and prognostic significance of PDGF ligands and receptors across soft tissue sarcomas. *ESMO open*, *6*(1), 100037. 10.1016/j.esmoop.2020.100037 10.1016/j.esmoop.2020.100037 PMC7848659 33524869

[118] Suzuki, S., Dobashi, Y., Hatakeyama, Y., Tajiri, R., Fujimura, T., Heldin, C. H., & Ooi, A. (2010). Clinicopathological significance of platelet-derived growth factor (PDGF)-B and vascular endothelial growth factor-A expression, PDGF receptor-β phosphorylation, and microvessel density in gastric cancer. *BMC Cancer,**10*, 659. 10.1186/1471-2407-10-659 21118571 10.1186/1471-2407-10-659 PMC3009982

[119] Paulsson, J., Lindh, M. B., Jarvius, M., Puputti, M., Nistér, M., Nupponen, N. N., … Hasselblatt, M. (2011). Prognostic but not predictive role of platelet-derived growth factor receptors in patients with recurrent glioblastoma. *International Journal of Cancer*, *128*(8), 1981–1988. 10.1002/ijc.25528 10.1002/ijc.25528 20589679

[120] Koos, B., Paulsson, J., Jarvius, M., Sanchez, B. C., Wrede, B., Mertsch, S., … Hasselblatt, M. (2009). Platelet-derived growth factor receptor expression and activation in choroid plexus tumors. *The American Journal of Pathology*, *175*(4), 1631–1637. 10.2353/ajpath.2009.081022 10.2353/ajpath.2009.081022 PMC2751559 19717644

[121] Jarvius, M., Paulsson, J., Weibrecht, I., Leuchowius, K.-J., Andersson, A.-C., Wählby, C., … Söderberg, O. (2007). In situ detection of phosphorylated platelet-derived growth factor receptor beta using a generalized proximity ligation method. *Molecular & Cellular Proteomics: MCP*, *6*(9), 1500–1509. 10.1074/mcp.M700166-MCP200 10.1074/mcp.M700166-MCP200 17565975

[122] Wåhlén, E., Olsson, F., Söderberg, O., Lennartsson, J., & Heldin, J. (2022). Differential impact of lipid raft depletion on platelet-derived growth factor (PDGF)-induced ERK1/2 MAP-kinase. *SRC and AKT Signaling. Cellular Signalling,**96*, 110356. 10.1016/j.cellsig.2022.110356 35605761 10.1016/j.cellsig.2022.110356

[123] Weigel, M. T., Dahmke, L., Schem, C., Bauerschlag, D. O., Weber, K., Niehoff, P., … Mundhenke, C. (2010). In vitro effects of imatinib mesylate on radiosensitivity and chemosensitivity of breast cancer cells. *BMC Cancer*, *10*(1), 412. 10.1186/1471-2407-10-412 10.1186/1471-2407-10-412 PMC2925350 20691121

[124] Kadivar, A., Ibrahim Noordin, M., Aditya, A., Kamalidehghan, B., Davoudi, E. T., Sedghi, R., & Akbari Javar, H. (2018). Antiproliferative effects of imatinib mesylate on ZR-75-1 and MDA-MB-231 cell lines via PDGFR-β, PDGF-BB, c-Kit and SCF expression. *International Journal of Molecular Medicine,**42*(1), 414–424. 10.3892/ijmm.2018.3590 29620139 10.3892/ijmm.2018.3590

[125] Pinto, M. P., Dye, W. W., Jacobsen, B. M., & Horwitz, K. B. (2014). Malignant stroma increases luminal breast cancer cell proliferation and angiogenesis through platelet-derived growth factor signaling. *BMC Cancer,**14*, 735. 10.1186/1471-2407-14-735 25274034 10.1186/1471-2407-14-735 PMC4190420

[126] Kuzmanov, A., Hopfer, U., Marti, P., Meyer-Schaller, N., Yilmaz, M., & Christofori, G. (2014). LIM-homeobox gene 2 promotes tumor growth and metastasis by inducing autocrine and paracrine PDGF-B signaling. *Molecular Oncology,**8*(2), 401–416. 10.1016/j.molonc.2013.12.009 24423492 10.1016/j.molonc.2013.12.009 PMC5528541

[127] Lev, D. C., Kim, S. J., Onn, A., Stone, V., Nam, D.-H., Yazici, S., … Price, J. E. (2005). Inhibition of platelet-derived growth factor receptor signaling restricts the growth of human breast cancer in the bone of nude mice. *Clinical Cancer Research: An Official Journal of the American Association for Cancer Research*, *11*(1), 306–314. 15671560

[128] Papadopoulos, N., & Lennartsson, J. (2018). The PDGF/PDGFR pathway as a drug target. *Molecular Aspects of Medicine,**62*, 75–88. 10.1016/j.mam.2017.11.007 29137923 10.1016/j.mam.2017.11.007

[129] Criscitiello, C., Gelao, L., Viale, G., Esposito, A., & Curigliano, G. (2014). Investigational platelet-derived growth factor receptor kinase inhibitors in breast cancer therapy. *Expert Opinion on Investigational Drugs,**23*(5), 599–610. 10.1517/13543784.2014.895323 24597540 10.1517/13543784.2014.895323

[130] Ehnman, M., & Östman, A. (2014). Therapeutic targeting of platelet-derived growth factor receptors in solid tumors. *Expert Opinion on Investigational Drugs,**23*(2), 211–226. 10.1517/13543784.2014.847086 24206431 10.1517/13543784.2014.847086

[131] Yam, C., Murthy, R. K., Rauch, G. M., Murray, J. L., Walters, R. S., Valero, V., … Arun, B. (2018). A phase II study of imatinib mesylate and letrozole in patients with hormone receptor-positive metastatic breast cancer expressing c-kit or PDGFR-β. *Investigational New Drugs*, *36*(6), 1103–1109. 10.1007/s10637-018-0672-z 10.1007/s10637-018-0672-z 30311036

[132] Tsioumpekou, M., Cunha, S. I., Ma, H., Åhgren, A., Cedervall, J., Olsson, A.-K., … Lennartsson, J. (2020). Specific targeting of PDGFRβ in the stroma inhibits growth and angiogenesis in tumors with high PDGF-BB expression. *Theranostics*, *10*(3), 1122–1135. 10.7150/thno.37851 10.7150/thno.37851 PMC6956815 31938055

[133] Thijssen, V. L., Paulis, Y. W., Nowak‐Sliwinska, P., Deumelandt, K. L., Hosaka, K., Soetekouw, P. M., … Griffioen, A. W. (2018). Targeting PDGF‐mediated recruitment of pericytes blocks vascular mimicry and tumor growth. *The Journal of Pathology*, *246*(4), 447–458. 10.1002/path.5152 10.1002/path.5152 PMC6587443 30101525

[134] Chen, L., Jiang, Y.-Z., Wu, S.-Y., Wu, J., Di, G.-H., Liu, G.-Y., … Shao, Z.-M. (2022). Famitinib with camrelizumab and nab-paclitaxel for advanced immunomodulatory triple-negative breast cancer (FUTURE-C-Plus): An open-label, single-arm, phase II trial. *Clinical Cancer Research: An Official Journal of the American Association for Cancer Research*, *28*(13), 2807–2817. 10.1158/1078-0432.CCR-21-4313 10.1158/1078-0432.CCR-21-4313 PMC9365373 35247906

[135] Klug, L. R., & Heinrich, M. C. (2017). PDGFRA antibody for soft tissue sarcoma. *Cell,**168*(4), 555. 10.1016/j.cell.2017.01.028 28187274 10.1016/j.cell.2017.01.028

[136] Liang, M., Wang, B., Schneider, A., Vainshtein, I., & Roskos, L. (2020). A novel pharmacodynamic biomarker and mechanistic modeling facilitate the development of tovetumab, a monoclonal antibody directed against platelet-derived growth factor receptor alpha, for cancer therapy. *The AAPS Journal,**23*(1), 4. 10.1208/s12248-020-00523-3 33210183 10.1208/s12248-020-00523-3

[137] Tap, W. D., Jones, R. L., Van Tine, B. A., Chmielowski, B., Elias, A. D., Adkins, D., … Schwartz, G. K. (2016). Olaratumab and doxorubicin versus doxorubicin alone for treatment of soft-tissue sarcoma: an open-label phase 1b and randomised phase 2 trial. *Lancet*, *388*(10043), 488–497. 10.1016/S0140-6736(16)30587-6 10.1016/S0140-6736(16)30587-6 PMC5647653 27291997

[138] Martín-Broto, J., Pousa, A. L., Brohl, A. S., Van Tine, B. A., Powers, B., Stacchiotti, S., … Jones, R. L. (2021). Circulating tumor cells and biomarker modulation with olaratumab monotherapy followed by olaratumab plus doxorubicin: Phase Ib study in patients with soft-tissue sarcoma. *Molecular Cancer Therapeutics*, *20*(1), 132–141. 10.1158/1535-7163.MCT-20-0441 10.1158/1535-7163.MCT-20-0441 33177152

[139] Tap, W. D., Wagner, A. J., Schöffski, P., Martin-Broto, J., Krarup-Hansen, A., Ganjoo, K. N., … ANNOUNCE Investigators. (2020). Effect of doxorubicin plus olaratumab vs doxorubicin plus placebo on survival in patients with advanced soft tissue sarcomas: The ANNOUNCE randomized clinical trial. *JAMA*, *323*(13), 1266–1276. 10.1001/jama.2020.1707 10.1001/jama.2020.1707 PMC7139275 32259228

[140] Camorani, S., Hill, B. S., Collina, F., Gargiulo, S., Napolitano, M., Cantile, M., … Cerchia, L. (2018). Targeted imaging and inhibition of triple-negative breast cancer metastases by a PDGFRβ aptamer. *Theranostics*, *8*(18), 5178–5199. 10.7150/thno.27798 10.7150/thno.27798 PMC6217067 30429893

[141] Camorani, S., Esposito, C. L., Rienzo, A., Catuogno, S., Iaboni, M., Condorelli, G., … Cerchia, L. (2014). Inhibition of receptor signaling and of glioblastoma-derived tumor growth by a novel PDGFRβ aptamer. *Molecular Therapy: The Journal of the American Society of Gene Therapy*, *22*(4), 828–841. 10.1038/mt.2013.300 10.1038/mt.2013.300 PMC3982505 24566984

[142] Camorani, S., Passariello, M., Agnello, L., Esposito, S., Collina, F., Cantile, M., … Cerchia, L. (2020). Aptamer targeted therapy potentiates immune checkpoint blockade in triple-negative breast cancer. *Journal of experimental & clinical cancer research: CR*, *39*(1), 180. 10.1186/s13046-020-01694-9 10.1186/s13046-020-01694-9 PMC7487859 32892748

[143] Gil Del Alcazar, C. R., Huh, S. J., Ekram, M. B., Trinh, A., Liu, L. L., Beca, F., … Polyak, K. (2017). Immune escape in breast cancer during in situ to invasive carcinoma transition. *Cancer Discovery*, *7*(10), 1098–1115. 10.1158/2159-8290.CD-17-0222 10.1158/2159-8290.CD-17-0222 PMC5628128 28652380

[144] Bai, F., Liu, S., Liu, X., Hollern, D. P., Scott, A., Wang, C., … Pei, X.-H. (2021). PDGFRβ is an essential therapeutic target for BRCA1-deficient mammary tumors. *Breast cancer research: BCR*, *23*(1), 10. 10.1186/s13058-021-01387-x 10.1186/s13058-021-01387-x PMC7819225 33478572

[145] Kaps, L., & Schuppan, D. (2020). Targeting cancer associated fibroblasts in liver fibrosis and liver cancer using nanocarriers. *Cells,**9*(9), E2027. 10.3390/cells9092027 10.3390/cells9092027 PMC7563527 32899119

[146] Tao, Z., Yang, H., Shi, Q., Fan, Q., Wan, L., & Lu, X. (2017). Targeted delivery to tumor-associated pericytes via an affibody with high affinity for PDGFRβ enhances the in vivo antitumor effects of human TRAIL. *Theranostics,**7*(8), 2261–2276. 10.7150/thno.19091 28740549 10.7150/thno.19091 PMC5505058

[147] Fan, J., Feng, Y., Tao, Z., Chen, J., Yang, H., Shi, Q., … Lu, X. (2021). A versatile platform for the tumor-targeted delivery of immune checkpoint-blocking immunoglobin G. *Journal of Controlled Release: Official Journal of the Controlled Release Society*, *340*, 243–258. 10.1016/j.jconrel.2021.11.003 10.1016/j.jconrel.2021.11.003 34752799

[148] Lindborg, M., Cortez, E., Höidén-Guthenberg, I., Gunneriusson, E., von Hage, E., Syud, F., … Frejd, F. Y. (2011). Engineered high-affinity affibody molecules targeting platelet-derived growth factor receptor β in vivo. *Journal of Molecular Biology*, *407*(2), 298–315. 10.1016/j.jmb.2011.01.033 10.1016/j.jmb.2011.01.033 21277312

[149] Wang, J.-C., Li, G.-Y., Wang, B., Han, S.-X., Sun, X., Jiang, Y.-N., … Liu, P.-J. (2019). Metformin inhibits metastatic breast cancer progression and improves chemosensitivity by inducing vessel normalization via PDGF-B downregulation. *Journal of experimental & clinical cancer research: CR*, *38*(1), 235. 10.1186/s13046-019-1211-2 10.1186/s13046-019-1211-2 PMC6549289 31164151

[150] Mao, X., Xu, J., Wang, W., Liang, C., Hua, J., Liu, J., … Shi, S. (2021). Crosstalk between cancer-associated fibroblasts and immune cells in the tumor microenvironment: New findings and future perspectives. *Molecular Cancer*, *20*(1), 131. 10.1186/s12943-021-01428-1 10.1186/s12943-021-01428-1 PMC8504100 34635121

[151] Barrett, R. L., & Puré, E. (n.d.). Cancer-associated fibroblasts and their influence on tumor immunity and immunotherapy. *eLife*, *9*, e57243. 10.7554/eLife.57243 10.7554/eLife.57243 PMC7769568 33370234

[152] Monteran, L., & Erez, N. (2019). The dark side of fibroblasts: Cancer-associated fibroblasts as mediators of immunosuppression in the tumor microenvironment. *Frontiers in Immunology,**10*, 1835. 10.3389/fimmu.2019.01835 31428105 10.3389/fimmu.2019.01835 PMC6688105

[153] Loh, J. J., & Ma, S. (2021). The role of cancer-associated fibroblast as a dynamic player in mediating cancer stemness in the tumor microenvironment. *Frontiers in Cell and Developmental Biology,**9*, 727640. 10.3389/fcell.2021.727640 34760886 10.3389/fcell.2021.727640 PMC8573407

[154] Ferguson, L. P., Diaz, E., & Reya, T. (2021). The role of the microenvironment and immune system in regulating stem cell fate in cancer. *Trends in Cancer,**7*(7), 624–634. 10.1016/j.trecan.2020.12.014 33509688 10.1016/j.trecan.2020.12.014 PMC8318571

[155] Appiah-Kubi, K., Lan, T., Wang, Y., Qian, H., Wu, M., Yao, X., … Chen, Y. (2017). Platelet-derived growth factor receptors (PDGFRs) fusion genes involvement in hematological malignancies. *Critical Reviews in Oncology/Hematology*, *109*, 20–34. 10.1016/j.critrevonc.2016.11.008 10.1016/j.critrevonc.2016.11.008 28010895

[156] Akanda, M. R., Ahn, E.-J., Kim, Y. J., Salam, S. M. A., Noh, M.-G., Lee, T.-K., … Moon, K.-S. (2023). Analysis of stromal PDGFR-β and α-SMA expression and their clinical relevance in brain metastases of breast cancer patients. *BMC cancer*, *23*(1), 468. 10.1186/s12885-023-10957-5 10.1186/s12885-023-10957-5 PMC10201734 37217880

